# BioTWavePat: An EEG-Based Mental Performance Detection Model

**DOI:** 10.3390/jcm15218471

**Published:** 2026-10-30

**Authors:** Tugce Keles, Mehmet Baygin, Irem Tasci, Erdem Gonultas, Taha Muezzinoglu, Burak Tasci, Sengul Dogan, Turker Tuncer

**Affiliations:** 1 Department of Digital Forensics Engineering, Technology Faculty, Firat University, 23119 Elazig, Türkiye; tkeles@firat.edu.tr (T.K.); turkertuncer@firat.edu.tr (T.T.); 2 Department of Computer Engineering, College of Engineering, Erzurum Technical University, 25050 Erzurum, Türkiye; mehmet.baygin@erzurum.edu.tr; 3 Department of Neurology, School of Medicine, Firat University, 23119 Elazig, Türkiye; itasci@firat.edu.tr; 4 Department of Computer Technologies, Bingöl University, 12000 Bingöl, Türkiye; egonultas@bingol.edu.tr; 5 Department of Computer Engineering, Faculty of Engineering, Munzur University, 62000 Tunceli, Türkiye; tahamuezzinoglu@munzur.edu.tr; 6 Department of Electrical and Energy, Vocational School of Technical Sciences, Firat University, 23119 Elazig, Türkiye

**Keywords:** mental performance detection, explainable feature engineering, Directed Lobish, rhythm transition features, leave-one-subject-out cross-validation

## Abstract

**Background/Objectives**: EEG-based mental performance classification requires participant-separated validation and traceable feature identities. **Methods**: BioTWavePat-XFE constructs five single-rhythm and 15 signed cross-rhythm channel-rank transition layers, uses four cumulative-weighted selectors and tkNN/IMV classification, and decodes retained identities with Directed Lobish. We compared Protocol A (whole-dataset feature and pooled-test outcome selection), Protocol B (training-fold outcome selection with Protocol A features), and Protocol C (all data-dependent steps restricted to outer training data). STEW and MAT used participant-wise LOSO. TMPD included all 55 participants in 54 identifier groups; two same-class participants sharing an identifier were withheld together. **Results**: Protocol A segment accuracies were 83.08% (STEW), 93.14% (TMPD), and 91.15% (MAT). Protocol C accuracies were 80.46%, 82.50%, and 74.65%, with balanced accuracies of 80.46%, 75.17%, and 64.15%, respectively. These are the primary estimates without the identified selection leakage under the stated participant-separated validation design. **Conclusions**: Whole-dataset selection inflated the original estimates, particularly for TMPD and MAT. MAT Protocol C performance was close to majority prediction. Directed Lobish exposes selected feature identities without establishing physiological connectivity, causality or biomarker validity.

## 1. Introduction

Mental performance detection is a commonly used process in education, cognitive assessment, neuroergonomics, and human–computer interaction [1,2]. Mental performance can change according to attention, workload, fatigue, motivation, and task difficulty [3]. These changes can affect learning quality, decision speed, response accuracy, and task success [4]. Therefore, automatic mental performance detection has become an important research topic.

Electroencephalography is one of the most useful signal sources for mental performance detection [5]. EEG records brain electrical activity with high temporal resolution. It can show fast neural changes during cognitive tasks [5]. EEG rhythms, such as delta, theta, alpha, beta, and gamma, can also reflect different brain states [6]. For this reason, EEG has been widely used for attention analysis, workload detection, fatigue estimation, and cognitive performance classification [7]. However, the brain structure related to mental performance is not fully known. Mental performance is not generated by a single brain region or a single EEG rhythm. It is related to distributed neural activity, rhythm changes, and interactions between brain regions [8]. Therefore, EEG-based mental performance detection remains a difficult problem. Subject variability, noise, artifact effects, and task differences can also reduce classification performance [9].

In the literature, several EEG-based models have been proposed for mental performance detection. Many studies have used statistical features, spectral features, connectivity measures, machine learning models, and deep learning architectures [10]. These models have reported promising classification results [11]. However, most existing models have mainly focused on classification accuracy. The explanation part has generally been limited [12]. Therefore, it is still difficult to understand which EEG rhythm layers, channel labels, or model-derived channel-pair features are selected for mental performance detection.

Explainable models are needed for this problem. In education and cognitive monitoring, a model should not only classify the mental state. It should also show meaningful information about the brain signal. For example, a traceable representation can identify the rhythm layer and ordered channel labels associated with each retained feature. Such identity decoding should be distinguished from a validated estimate of physiological or predictive importance. This type of explanation can increase the clinical, cognitive, and educational value of the model [13].

To address this need, a new explainable feature engineering model is proposed in this research. The proposed model is named Biological Turker Wave Pattern Explainable Feature Engineering (BioTWavePat-XFE). The model uses BioTWavePat for rhythm-based feature extraction. BioTWavePat generates transition tables from EEG rhythm layers. These layers include single-rhythm maps and directional cross-rhythm maps. Therefore, the model can extract both within-rhythm and rhythm-to-rhythm transition information.

In the proposed model, feature selection is performed using four selectors: neighborhood component analysis (NCA) [14], chi-square (Chi2) [15], minimum redundancy maximum relevance (mRMR) [16], and ReliefF [17]. These selectors are used to choose informative features from the extracted BioTWavePat feature space. Then, k-nearest neighbor classifiers are used with different distance and weighting settings. Improved majority voting is applied to combine strong prediction outputs. This classification model is termed the t-algorithm-based kNN (tkNN) model [18]. Finally, Directed Lobish [19] is used to generate explainable results from selected features. Directed Lobish is an important part of the proposed model. Each selected BioTWavePat feature has a rhythm-layer identity, a source-channel identity, and a target-channel identity. Therefore, selected features can be converted into directed symbolic explanations. These symbolic summaries identify frequently retained rhythm-layer and ordered channel-label identities. Thus, the model provides classification outputs and representation-level traceability.

We evaluated BioTWavePat-XFE separately on STEW [20], MAT [21], and TMPD [22]. Protocol A segment accuracies were 83.08%, 91.15%, and 93.14%, respectively. Corresponding Protocol C accuracies were 80.46%, 74.65%, and 82.50%, with the held-out participant excluded from all data-dependent steps. These separate within-dataset evaluations do not demonstrate frozen-model transfer across devices, tasks, or datasets. TMPD used 54 identifier-group partitions covering all 55 participants, with the merged same-class pair withheld together.

### 1.1. Related Work

EEG-based mental performance, mental workload, and cognitive load detection have been widely investigated using signal processing, feature engineering, connectivity analysis, machine learning, and deep learning models [23,24]. Existing studies have generally used spectral features, statistical descriptors, time-frequency representations, effective or functional connectivity measures, graph-based representations, recurrent neural networks, convolutional neural networks, transformers, and ensemble classifiers [25]. These studies have reported promising classification performance on benchmark datasets such as STEW [20] and MAT [21], as well as on task-specific cognitive workload datasets. However, most existing models have mainly focused on classification accuracy. Their explainable outputs are generally limited, especially in terms of rhythm-level, channel-level, and directed channel-transition-based interpretation [26]. Moreover, many deep learning-based models have complex architectures and limited traceability, while several feature engineering-based methods use fixed feature representations without explicitly modeling rhythm-to-rhythm and channel-to-channel transition patterns [27]. Therefore, an EEG-based mental performance detection model that combines high classification performance, subject-level generalization, and traceable explanations of selected feature identities is still needed [28]. Some representative studies in the literature are summarized in Table 1.

Although the studies summarized in Table 1 achieved promising classification results, several limitations can be observed. Connectivity-based approaches have provided useful information about brain-region interactions. For example, Maghsoudi and Shalbaf [29] used effective connectivity measures for mental arithmetic task recognition, while Safari et al. [8] investigated brain connectivity patterns for mental workload classification. Similarly, Gangapuram and Manian [34] used functional connectivity with a graph-based learning model. However, these studies did not explicitly convert the selected features into rhythm-layer and directed channel-transition rules.

Deep learning-based models have also been widely used in EEG-based workload and mental performance detection. Siddhad et al. [31] used transformer networks for raw EEG classification, and Ajra et al. [32] proposed a CNN-based model using spectral-temporal EEG features. Nguyen et al. [33] combined time-domain and frequency-domain deep representations for mental workload estimation. In addition, Afzal et al. [35] employed a deep gated neural network, while Siddhad et al. [36] used a ConvNeXt-based architecture. Although these models achieved strong classification performance, their architectures are generally more complex, and their decision mechanisms are less traceable than feature engineering-based approaches.

Some studies have also used multimodal or optimization-based strategies. Aksu et al. [13] combined EEG and eye-tracking features for mental workload assessment. Yedukondalu et al. [11] used lead-wise feature optimization and ensemble classification on MAT and STEW datasets. These approaches improved classification performance, but they did not mainly focus on model-intrinsic rhythm-channel explanation. In addition, several previous studies were evaluated on a single dataset or under task-specific settings. Therefore, there is still a need for a traceable and explainable EEG feature engineering model that can jointly provide high classification performance, LOSO-based subject-level generalization, rhythm-level interpretation, and directed channel-transition-based explanation across multiple mental performance datasets.

### 1.2. Literature Gaps

- In EEG-based mental performance detection, many models have focused on accuracy. However, interpretable result generation has remained limited.
- Several approaches offer rhythm-level or channel-level summaries, but the granularity and validation of these explanations vary. Traceable feature identities should be distinguished from validated effects on predictions or physiology.
- Deep learning models have been widely used in EEG classification. These models can give high accuracy. However, they are often complex and less explainable.
- Feature engineering models can expose the construction of individual descriptors. Their computational cost still depends on representation size, feature selection, classifier implementation, and validation design.
- Many feature engineering models use fixed and static features. However, EEG mental performance patterns can change across time, rhythm, and subject.
- Many studies have used only one dataset. Therefore, their performance on different mental performance datasets is not clearly known.
- Some studies have used segment-level validation. This can increase subject-specific bias. Therefore, LOSO CV is needed for stronger subject-level testing.
- Most studies do not provide both high classification performance and clear explanations. Therefore, an explainable and accurate EEG feature engineering model is still needed.

### 1.3. Motivation and Our Model

Mental performance detection is an important task for education and cognitive monitoring [37]. However, mental performance is not related to only one EEG rhythm or one brain region. It is related to rhythm changes, channel interactions, and subject-specific brain activity [38,39]. Therefore, a model should analyze EEG signals in a more meaningful and traceable way.

In many studies, EEG-based mental performance models have mainly aimed to increase classification accuracy. However, high accuracy alone is not sufficient. The model should also explain which rhythm layers and channel-pair feature identities are selected. Such traceability may support inspection of a representation intended for educational or cognitive assessment research [40].

Deep learning models can provide high performance in EEG classification [41]. Computational requirements depend on architecture, input size, and implementation. Their decision mechanisms may also require additional interpretation [42]. This motivates a traceable feature engineering alternative; lower computational cost requires a matched empirical comparison.

Feature engineering models can retain explicit descriptor identities. Many existing feature engineering models use fixed features. These static features may not fully represent mental performance-related EEG changes [43]. EEG mental performance patterns can change across rhythms, channels, and subjects [44]. Therefore, a rhythm-transition-based feature extraction model is needed. Participant separation is necessary for EEG generalization testing [45]. Participant-held-out testing has been used for workload classification [46]. We distinguish the original whole-dataset selection analysis from reruns restricting outcome selection, or all data-dependent steps, to the outer training data. Holding out an identifier alone does not prevent feature-selection leakage; the preprocessing and selection boundary must also be respected.

BioTWavePat-XFE combines single-rhythm and signed cross-rhythm transition features, tkNN classification, and Directed Lobish identity decoding. The evaluation examines the effect of the selection protocol and the traceability of retained features.

### 1.4. Innovations and Contributions

#### 1.4.1. Novelties

- BioTWavePat-XFE extends earlier pattern-based EEG frameworks with a rhythm-indexed feature representation; it does not introduce a new feature selector, classifier, or symbolic language.
- The extractor combines five band-filtered EEG maps and 15 signed cross-rhythm maps with normalized channel-rank transition tables. The stored feature vector contains 20C^2^ entries for C channels, each retaining a layer and an ordered channel-pair identity.
- Cross-rhythm maps are computational differences between filtered signals. The implemented bank covers 10 unordered rhythm pairs, with both signs retained for five pairs, as specified in Equations (6)–(11). Opposite-sign maps are algebraically related; the 15 difference maps are not claimed to be independent rhythm interactions or physiological information flow.
- NCA, Chi2, mRMR, ReliefF, tkNN, majority voting, and Directed Lobish are established supporting components. Their use together is an implementation choice; the novelty claim is restricted to the specified rhythm-layer representation and its feature-identity decoding.

#### 1.4.2. Contributions

- We evaluated BioTWavePat-XFE on three distinct EEG datasets. Protocol A segment accuracies were 83.08% (STEW), 91.15% (MAT), and 93.14% (TMPD). Protocol C, which excludes the test participant from every data-dependent step, obtained 80.46%, 74.65%, and 82.50%, respectively. Reporting both result sets exposes selection bias while retaining the original analysis for transparency. TMPD used 54 identifier-group partitions covering all 55 participants, with the merged same-class pair withheld together.

### 1.5. Novelty Boundary Relative to Prior Pattern Methods

Channel-rank coding, transition tables, and symbolic feature decoding predate BioTWavePat. ChannelPat with Lobish [18] encodes ranked channel pairs, TTPat [19] uses channel-based transition features and introduces Directed Lobish, and CubicPat [22] encodes ranked channel triplets. MountPat [47] constructs five sample-vector maps and 10 pair-difference maps; DiffPat [48] counts changed rank positions between consecutive sample vectors. Appendix A.3 Table A7 provides a dedicated comparison of these constructions and their stored feature dimensions. BioTWavePat uses a filtered rhythm bank before rank-transition counting and retains the rhythm-layer identity in the decoded feature.

For C channels, the stored dimensions are C^2^ for ChannelPat and DiffPat, 15C^2^ for MountPat, C^3^ for CubicPat, and 20C^2^ for BioTWavePat. TTPat reports 392 features for 14 channels; a general channel-count formula is not inferred from this reported configuration. BioTWavePat therefore stores 33.33% more entries than MountPat. Relative to CubicPat, its dimension ratio is 20/C: 3920 versus 2744 entries at C = 14 and 20,480 versus 32,768 at C = 32. These are representation-size comparisons, not measurements of information gain, execution time, or classification benefit. The signed difference bank contains 10 unordered pair families and five additional opposite-sign orientations; algebraic dependence between these maps limits an independence interpretation.

We report outcome-selection and fully outer-fold-internal reruns alongside the original analysis. Appendix A.6 Table A11 presents rhythm/channel ablations (T06), and Appendix A.6 Table A15 compares TTPat, LobishPat, CubicPat, MountPat, and DiffPat (T13). Both analyses used Protocol A. Their BioTWavePat references were 83.08% (STEW), 93.14% (TMPD), and 91.15% (MAT). These comparisons describe the original selection setting and do not establish an extractor or component advantage under Protocol C.

## 2. Dataset

In this study, three EEG-based mental performance datasets were used to evaluate the proposed BioTWavePat-XFE model. These datasets were STEW [20], TMPD [22], and MAT [21]. The datasets were selected because they represent different mental performance and cognitive workload conditions. STEW contains EEG recordings collected during a multitasking workload experiment. MAT contains EEG recordings obtained before and during a mental arithmetic task. TMPD contains EEG segments collected during a mental capacity test. Therefore, the proposed model was evaluated on heterogeneous EEG mental performance data rather than on a single experimental setting.

The general characteristics of the datasets are summarized in Table 2. These datasets differ in terms of task design, number of EEG channels, sampling frequency, recording protocol, and class definition. This diversity is important because it allows the proposed model to be tested under different cognitive task conditions.

As shown in Table 2, STEW concerns multitasking-induced workload, MAT concerns arithmetic performance, and TMPD uses a mental-test-score threshold. The datasets support separate evaluations of the same feature-construction framework under heterogeneous settings. Separate within-dataset LOSO results do not establish that a model trained in one setting generalizes unchanged to another.

STEW labels were assigned at participant level by a binary split of self-reported workload scores, yielding 24 low and 24 high participants. All 74 segments from each participant shared that participant’s reference label. MAT used 19 EEG channels from the standard 10–20 system. TMPD included all 55 participants under 54 recorded identifiers. Two participants from the same reference class shared one identifier and were held out together; the 54 identifier groups comprised 37 low and 17 high groups. No participant was excluded.

The three datasets were analyzed separately under Protocols A, B, and C. The outer held-out unit was one participant for STEW and MAT and one recorded identifier group for TMPD. TMPD contained 55 participants under 54 identifiers: two same-class participants shared one identifier and were withheld together in the same fold. All 55 participants were included; no participant was excluded. Thus, TMPD used 54 identifier-group-held-out folds rather than 55 one-person LOSO folds. Protocol C excludes the complete held-out unit from all data-dependent preprocessing, feature selection, and outcome selection. Separate within-dataset analyses do not establish frozen-model cross-dataset transfer.

Table 3 summarizes the segment-level observations. STEW contributed exactly 74 segments per participant. Segment counts varied between participants in TMPD and MAT. STEW and MAT decision aggregation was participant-based; TMPD aggregation was performed within the recorded identifier groups.

Table 3 shows balanced STEW segments and imbalanced TMPD and MAT segments. We report balanced accuracy, macro-F1 and both class recalls alongside accuracy. All segments of the outer held-out unit were excluded from classifier training. For TMPD, the two participants sharing an identifier were held out together. Protocols A and B still use whole-dataset feature selection; only Protocol C restricts every data-dependent step to the outer training data.

## 3. Methods

**Figure 1:**
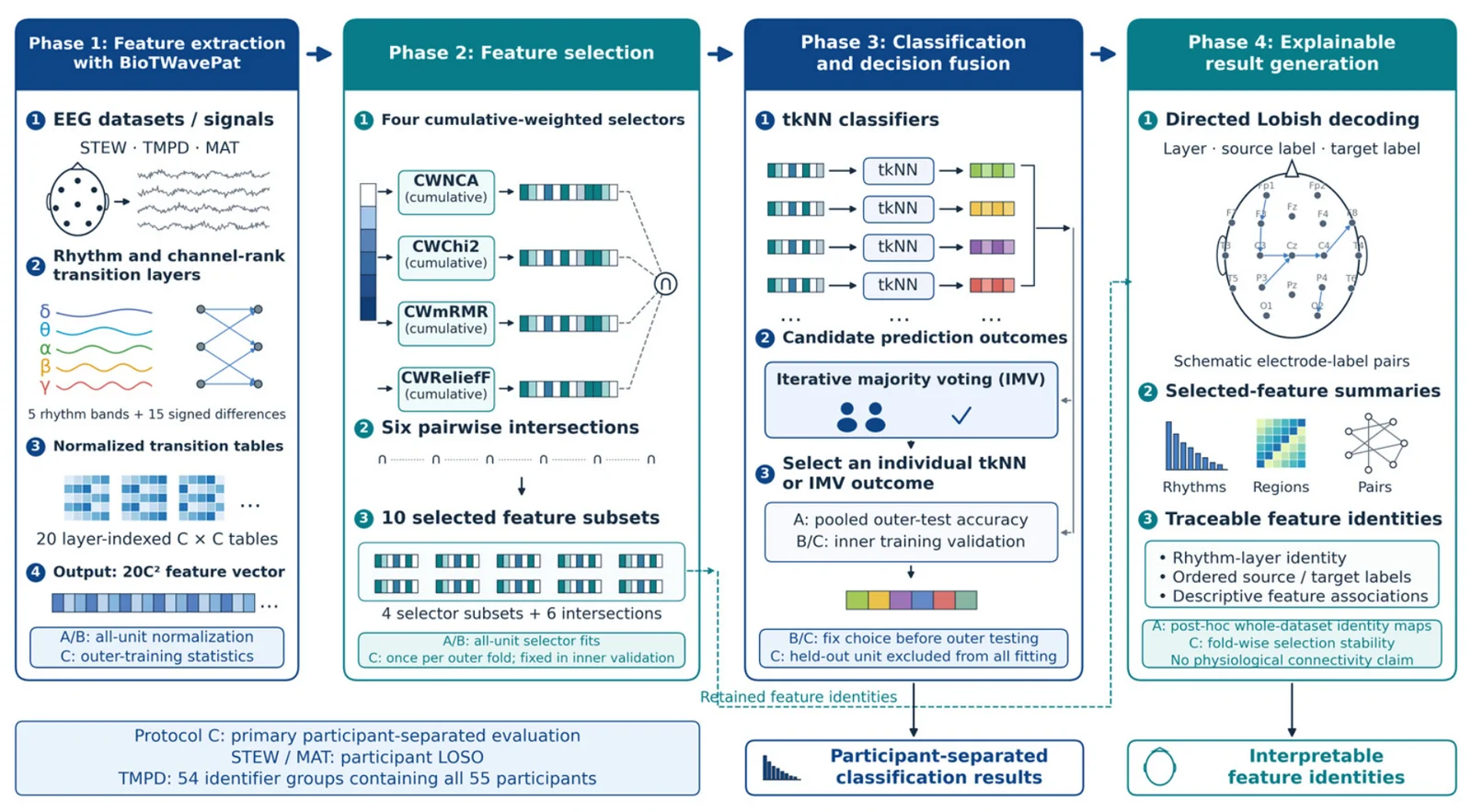
BioTWavePat-XFE stages with protocol-specific feature and outcome selection. Protocol A post hoc identity maps and Protocol C fold-wise stability are distinguished.

The essential objective of this research is to obtain AI-based information from the EEG data while generating interpretable results achieving high classification performance. Therefore, this XFE architecture contains four main phases, and these are as follows:

- Feature extraction employs BioTWavePat. The introduced BioTWavePat contains rhythm and channel transitions. Therefore, this feature extraction function generates both interpretable and informative features.
- To select the most informative features, four cumulative weighted feature selectors are utilized. These feature selectors are NCA [14], Chi2 [15], mRMR [16], and ReliefF [17]. Therefore, these feature selection functions are termed CWNCA, CWChi2, CWmRMR, and CWReliefF. Moreover, six binary-intersection feature subsets were generated from the selected feature sets.
- tkNN [18] classifier is used to generate classification results. Moreover, iterative majority voting (IMV) is utilized to create voted results.
- Utilizing Directed Lobish [19], XAI results are generated.
- The general block diagram of the presented BioTWavePat-XFE is depicted in Figure 1.

Figure 1 summarizes the representation, selection, classification, and feature-identity decoding stages. 

### 3.1. Biological Turker Wave Pattern

**Figure 2:**
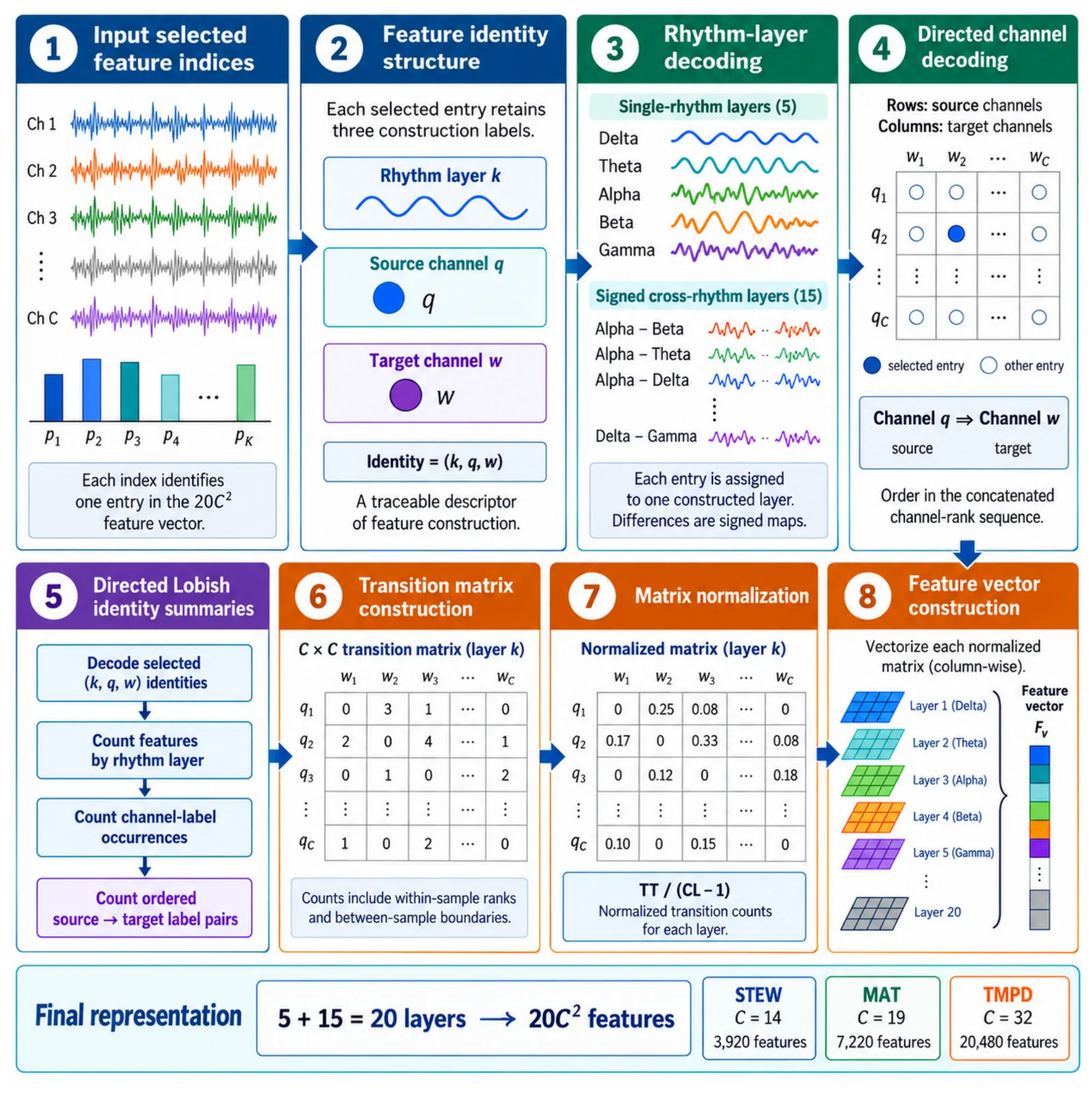
Block diagram of the presented BioTWavePat feature extraction function.

The objective of BioTWavePat is to extract rhythm-based and explainable transition features from multichannel EEG signals. BioTWavePat represents rhythm-layer values and ordered channel-index transitions. Here, direction is defined by the feature-construction sequence and does not establish neural information flow or connectivity. Therefore, it can generate informative features for classification and interpretable feature identities for explanation. The core innovation of this research is the proposed BioTWavePat extractor. Figure 2 shows its block diagram.

The explanation of the recommended BioTWavePat is given below.

Robust global normalization is applied. This normalization uses the median and median absolute deviation. Thus, the effect of extreme values is reduced.

For Protocol A, robust-normalization statistics and feature-wise min–max limits were estimated using all participants; these choices can transmit test-participant information. Protocol B retains this preprocessing and feature selection. For Protocol *C*, the median, median absolute deviation, and min–max limits were estimated only on the outer training participants and applied unchanged to the excluded participant.

The steps of the presented BioTWavePat are given below.

Step 1: Apply robust global normalization to input signal.

Step 2: Generate rhythm bands of the EEG signal.

(1) $$D=BRE\left(Sig,\left[0.5-4\;\mathrm{Hz}\right]\right)$$

(2) $$T=BRE\left(Sig,\left[4-8\;\mathrm{Hz}\right]\right)$$

(3) $$A=BRE\left(Sig,\left[8-13\;\mathrm{Hz}\right]\right)$$

(4) $$B=BRE\left(Sig,\left[13-30\;\mathrm{Hz}\right]\right)$$

(5) $$G=BRE\left(Sig,\left[>30\mathrm{Hz}\right]\right)$$

Herein, BRE(.): band rhythm extraction function, D: delta, T: theta, A: alpha, B: beta and G: gamma rhythms.

Step 3: Construct rhythm layer.

(6) $$L_{1}=A,\;L_{2}=B,\;L_{3}=G,\;L_{4}=T,\;L_{5}=D$$

(7) $$L_{6}=A-B,\;L_{7}=A-T,\;L_{8}=A-D$$

(8) $$L_{9}=G-A,\;L_{10}=G-B,\;L_{11}=G-T,\;L_{12}=G-D$$

(9) $$L_{13}=T-D,\;L_{14}=T-A,\;L_{15}=T-B$$

(10) $$L_{16}=B-A,\;L_{17}=B-T,\;L_{18}=B-G,\;L_{19}=B-D$$

(11) $$L_{20}=A-G$$

where L: the layer.

Step 4: Apply channel transformation to layers.

(12) $${Tr}_{k}\left(cnt:cnt+C-1\right)=argsort\left(-L_{k}\left(:,n\right)\right),\;k\;\in \;\{1,\;2,\;\ldots ,\;20\},\;n\;\in \;\{1,\;2,\;\ldots ,\;L\}\;cnt=1+(n-1)C$$

Here, Tr: the transformed signal, C: number of channels, and L: length of the signal.

In Equation (12), *n* is the time-sample index (*n = 1, …, L*), and *cnt = 1 + (n − 1)C* is the one-based starting position of the corresponding *C*-element block in *Tr_k_*. At each time sample, *argsort(−L_k_(:, n))* returns the channel indices in descending order of their layer values. These indices are concatenated in temporal order, giving Tr_k_ a length of CL. Thus, cnt takes the values *1, C + 1, 2C + 1, …, (L − 1)C + 1* and is reset to *1* for each layer *k*.

For samples without tied layer values, sign reversal reverses the channel ranking. The within-sample transition counts of opposite-sign layers *(L14, L16, L17, L18, L20)* are then the transpose of those of their paired layers *(L7, L6, L15, L10, L9)*. Sample-block boundaries can differ. We used MATLAB(2023b) sort with stable tie handling: equal values retain their original channel order. Consequently, tied samples can also violate the transpose relationship. Opposite-sign layers remain algebraically dependent, and a decoded direction is not unique across a sign-flipped pair.

Step 5: Fill the transition tables using transformed signals.

For each layer *k*, a *C × C* transition-count matrix *TT_k_* is initialized to zero. Its row index denotes the source channel, and its column index denotes the target channel.

(13) $${TT}_{k}(q,\;w)\;\leftarrow \;{TT}_{k}(q,\;w)+1,\;q={\mathrm{Tr}}_{k}(j),\;w={\mathrm{Tr}}_{k}(j+1),\;j\in \{1,2,\ldots ,\mathrm{CL}-1\}$$

The update in Equation (13) is applied for *j = 1, …, CL − 1* to the *CL*-element channel-index sequence *Tr_k_*. Each adjacent pair contributes one count, including pairs at the boundaries between consecutive *C*-element blocks.

Step 6: Repeat Steps 2–5 to fill all transition tables.

Step 7: Normalize transition table.

(14) $${{TT}_{k}}^{N}(q,\;w)=\frac{{TT}_{k}(q,\;w)}{S_{k}}\;S_{k}=\sum_{q=1}^{C}\sum_{w=1}^{C}{\mathrm{TT}}_{k}(q,w)=\mathrm{CL}-1$$

In Equation (14), *S_k_* is the sum of all *C*^2^ entries of *TT_k_*. Because Equation (13) counts every adjacent pair in a sequence of length *CL, S_k_ = CL − 1*. The normalized table *TT_k_* retains the *C × C* dimensions, has nonnegative entries, and sums to one over all entries.

Step 8: Deploy tensor to vector transformation and concatenate vectors to obtain final feature vector.

(15) $${vek}_{k}(a)={{TT}_{k}}^{N}(q,\;w),\;a=q+(w-1)C,\;q,w\in \{1,2,\ldots ,C\}$$

(16) $$Fv(a+C^{2}(k-1))={vek}_{k}(a),\;a\in \{1,2,\ldots ,C^{2}\},\;k\in \{1,2,\ldots ,20\}$$

Equation (15) uses column-wise vectorization, consistent with MATLAB *TT_k_(:)*. The row index *q* varies first within each column w, so *a = q + (w − 1)C*. Thus, entries from column 1 precede those from column 2, and so on. Each vector vek_k_ contains *C*^2^ entries. Equation (16) concatenates these vectors in layer order *k = 1, …, 20*, placing layer k at indices *(k − 1)C*^2^ *+ 1* through kC^2^ in *Fv*. The resulting feature vector contains *20C*^2^ entries.

The feature length follows directly from the layer and table dimensions. Equations (6)–(11) define five single-rhythm layers and 15 cross-rhythm layers, giving 20 layers in total. Each layer contributes a complete *C × C* transition table. Column-wise vectorization retains all *C*^2^ matrix positions, including diagonal entries and zero-count entries. Therefore, the number of entries d in the concatenated feature vector is

$$d=\sum_{k=1}^{20}C^{2}=(5+15)C^{2}=20C^{2}$$

This is the stored vector length; normalization changes the feature values but does not remove entries. In Equation (16), layer k occupies the *C*^2^ consecutive positions from (*k −* 1)*C*^2^ *+* 1 *to kC*^2^. The 20 blocks are contiguous and nonoverlapping: the first index is 1, and the final index is *C*^2^ *+ C*^2^(20 − 1) *=* 20*C*^2^. This index calculation independently verifies the concatenated feature length.

### 3.2. Feature Selection

**Figure 3:**
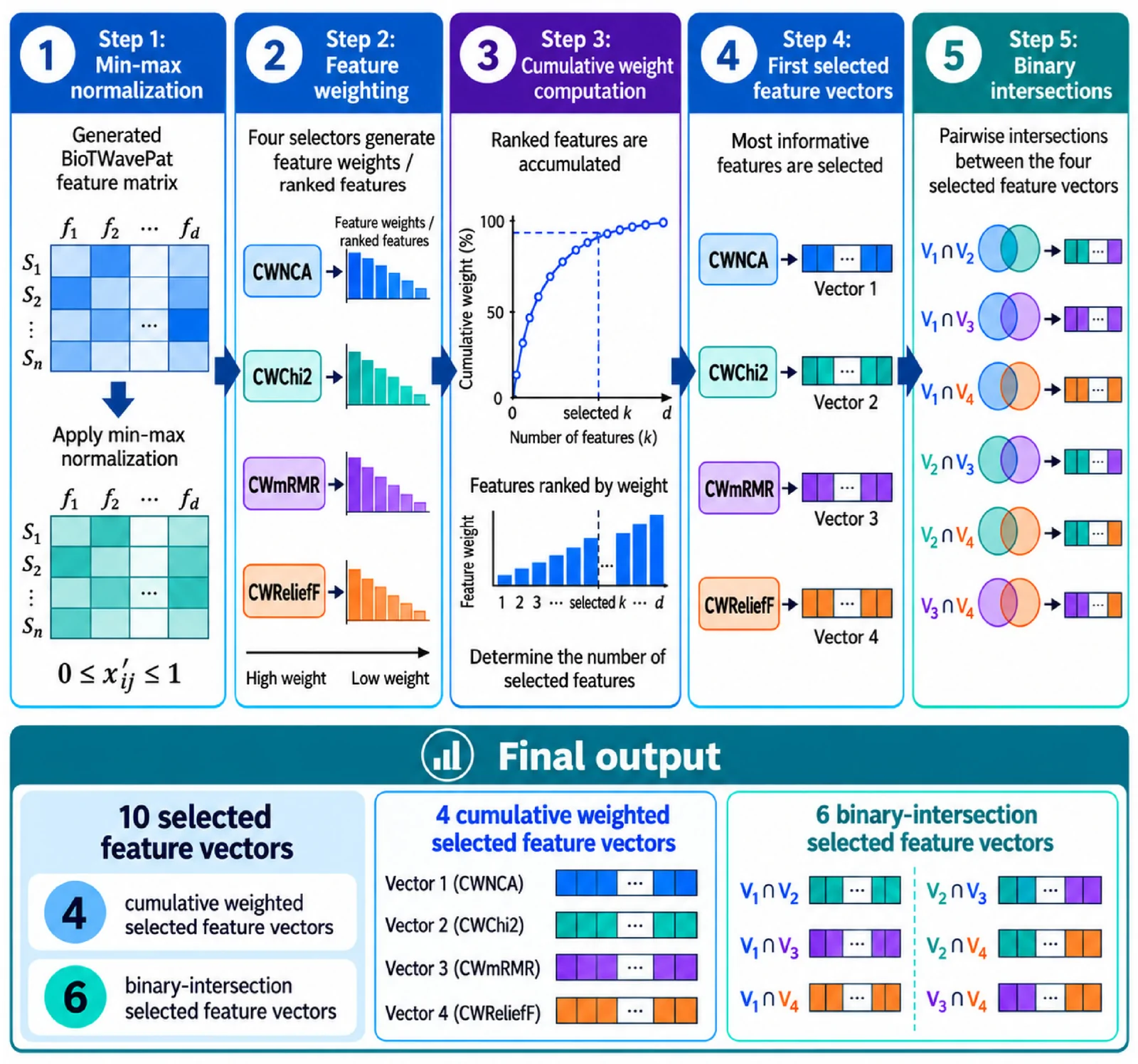
Graphical illustration of the presented cumulative weighted multiple feature selection. Herein, CW: Cumulative Weight, V: selected feature vector.

In the feature selection phase, four cumulative weighted feature selectors were used. These selectors were CWNCA, CWChi2, CWmRMR, and CWReliefF. The aim of this phase was to select the most informative BioTWavePat features before classification. BioTWavePat generates a high-dimensional feature vector because it uses 20 rhythm layers and channel-to-channel transition tables. Therefore, feature selection was needed to remove weak, noisy, and redundant features. The depiction of the presented feature selection is illustrated in Figure 3.

The steps of the proposed multiple cumulative weighted feature selection method are given below.

Step 1: Apply min-max normalization to the generated feature matrix.

Step 2: Generate feature weights using NCA [14], Chi2 [15], mRMR [16], and ReliefF [17].

Step 3: Apply cumulative weight computation and determine the number of selected features.

Step 4: Select the most informative features and generate the first four selected feature vectors.

Step 5: Apply binary intersection and generate six additional selected feature vectors.

Finally, ten selected feature vectors were generated in this phase. Four of them were obtained from the cumulative weighted selectors. The remaining six were obtained from the binary intersections of these selected feature vectors.

#### 3.2.1. Protocol A—Whole-Dataset Feature Selection

In Protocol A, normalization and feature selection were performed once using the complete dataset. The four cumulative-weighted selectors (CWNCA, CWChi2, CWmRMR, and CWReliefF) were applied to all participants. Their feature weights were used to determine the cumulative thresholds and retain the highest-ranked features. Six additional subsets were obtained from pairwise intersections of the four selected feature sets, yielding ten feature subsets in total. These subsets were kept fixed during classification. Because the held-out participants contributed to feature selection, this protocol retained information leakage. The corresponding classification procedure is described in Section 3.3.1.

#### 3.2.2. Protocol B—Reuse of Globally Selected Features

Protocol B used the same normalization parameters and selected feature subsets as Protocol A. No feature weights, thresholds, or intersections were recalculated within the outer training folds. The only change concerned the selection of the tkNN/IMV prediction outcome, which was based on inner validation within the outer training data, as described in Section 3.3.2. Thus, Protocol B removed test-based outcome selection but retained the feature-selection leakage introduced by the whole-dataset analysis.

#### 3.2.3. Protocol C— Training-Only Feature Selection Within Outer Folds

In Protocol C, normalization and feature selection were repeated independently for each outer training fold. The held-out participant, or identifier group in TMPD, was excluded from every fitting and selection step. The four cumulative-weighted selectors were fitted using only the outer training data. Feature weights, cumulative thresholds, retained indices, and six pairwise intersections were then recalculated for that fold. The resulting feature subsets remained fixed during inner validation, where tkNN/IMV outcomes were compared. Feature selectors were not refitted within individual inner splits. Therefore, Protocol C prevented outer-test information from entering feature selection, although the inner procedure did not constitute fully nested feature selection. Final performance was evaluated exclusively on the outer held-out units, as described in Section 3.3.3.

### 3.3. Classification

tkNN configurations classified the four selector-specific subsets and six binary intersections. IMV combined candidate prediction outputs. The selection boundary differed across the three protocols. Protocols B and C used inner outcome validation with a fixed feature space; neither performed fully nested refitting of feature selectors.

#### 3.3.1. Protocol A—Original Analysis

We selected the final individual tkNN or IMV outcome as the candidate with the highest pooled outer-test accuracy after whole-dataset normalization and feature selection. Thus, feature selection and outcome choice used all participants.

#### 3.3.2. Protocol B—Training-Fold Outcome Selection

We retained the whole-dataset normalization and selected feature indices of Protocol A. Individual tkNN and IMV outcomes were compared by accuracy in inner validation using only the outer-training data, with these Protocol A feature indices fixed. The highest-validation-accuracy outcome was fitted on the outer training data and fixed before test inference. This removes test-based outcome choice but retains leakage from whole-dataset feature selection; the feature sets themselves were not re-estimated from the outer training partition in Protocol B.

#### 3.3.3. Protocol C—Fully Fold-Internal Pipeline (Leakage-Free)

We re-estimated normalization, four cumulative-weighted selectors, cumulative thresholds, and intersections using only each outer training partition. Within each outer training partition, individual tkNN and IMV outcomes were compared by accuracy in an inner validation using only the outer-training data. The feature space was fixed from that outer training partition; feature selectors were not refitted within each inner validation split. The highest-validation-accuracy outcome was fixed before the outer test unit was evaluated. This is outer-fold-internal outcome selection, not fully nested feature selection. The excluded participant or identifier group did not contribute values or labels to any data-dependent stage. Protocol C avoids the identified outer-test selection leakage under the stated validation design.

Inner validation was used to choose the outcome, whereas final performance was computed only from predictions for the outer held-out units. Because feature selection was fixed from the outer training partition, inner validation scores are model-selection criteria, not independently validated performance estimates. The outer test unit remained excluded throughout.

**Figure 4:**
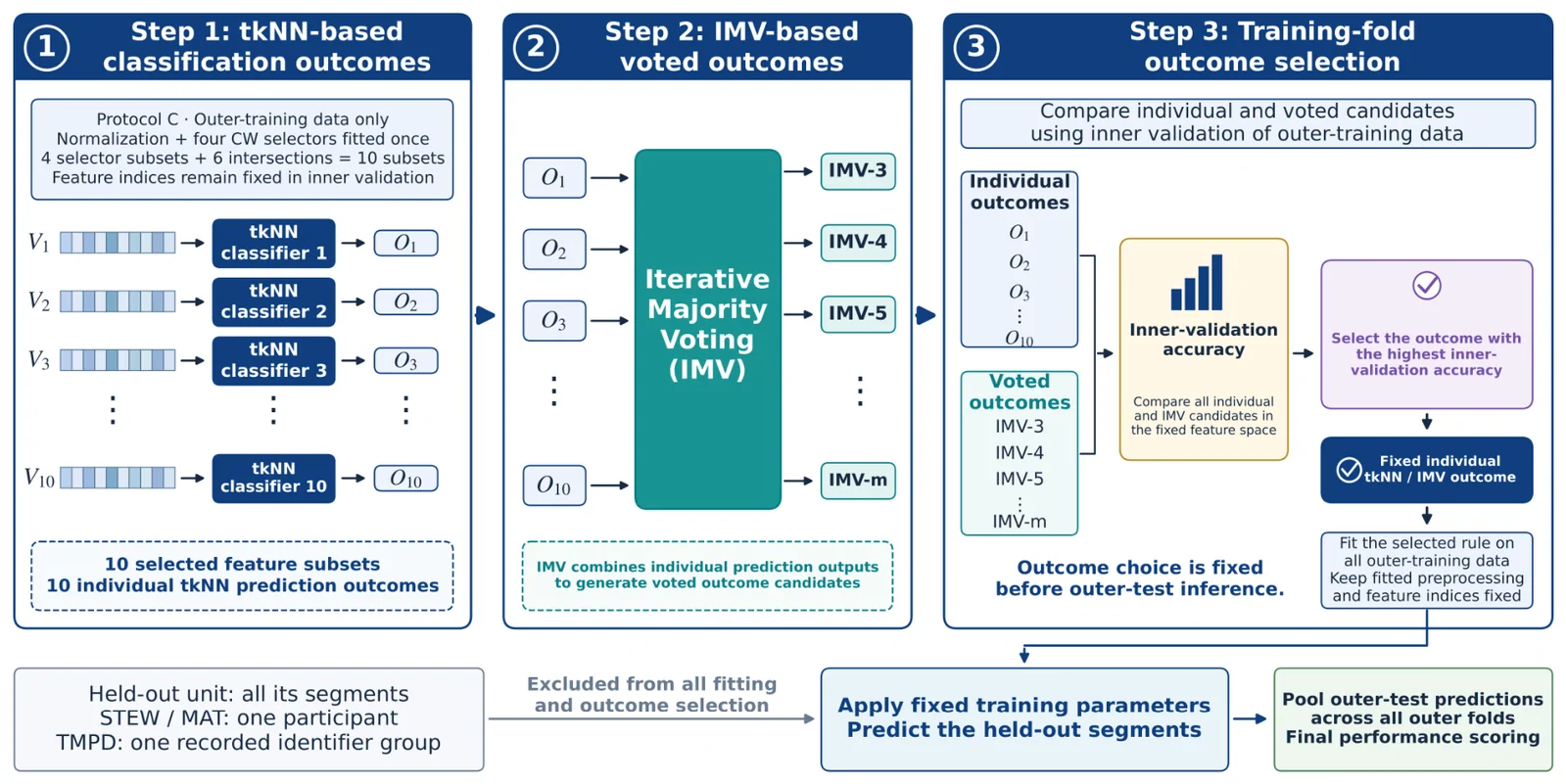
Protocol C outer validation fold. Selectors are fitted once on the outer training partition; a fixed feature space is used for inner outcome validation. The complete held-out participant or TMPD identifier group is excluded from all fitting and selection.

The flowchart of the tkNN-IMV classification phase is shown in Figure 4.

The steps of the tkNN-IMV classification phase are given below.

Step 1: Hold out all segments of one participant (STEW/MAT) or recorded identifier group (TMPD). Estimate normalization parameters, fit the four selectors and derive cumulative thresholds and intersections using only the remaining units.

Step 2: Compare individual tkNN and IMV outcomes by accuracy in inner validation using only the outer training data, with its selected feature space fixed. Fix the selected outcome before outer-test inference.

Step 3: Fit the chosen prediction rule on the outer training data with the fixed feature indices and preprocessing parameters. Predict the held-out unit and pool outer-test predictions for final scoring.

The data boundary for selection differs across Protocols A, B, and C, as specified in Section 3.2 and Section 3.3 and Figure 4.

Figure 4 separates inner outcome validation from outer performance estimation. The four selectors are fitted once per outer fold, rather than refitted within every inner validation split. Protocol A instead uses whole-dataset selection and pooled-test outcome choice.

### 3.4. XAI Results Generation

**Figure 5:**
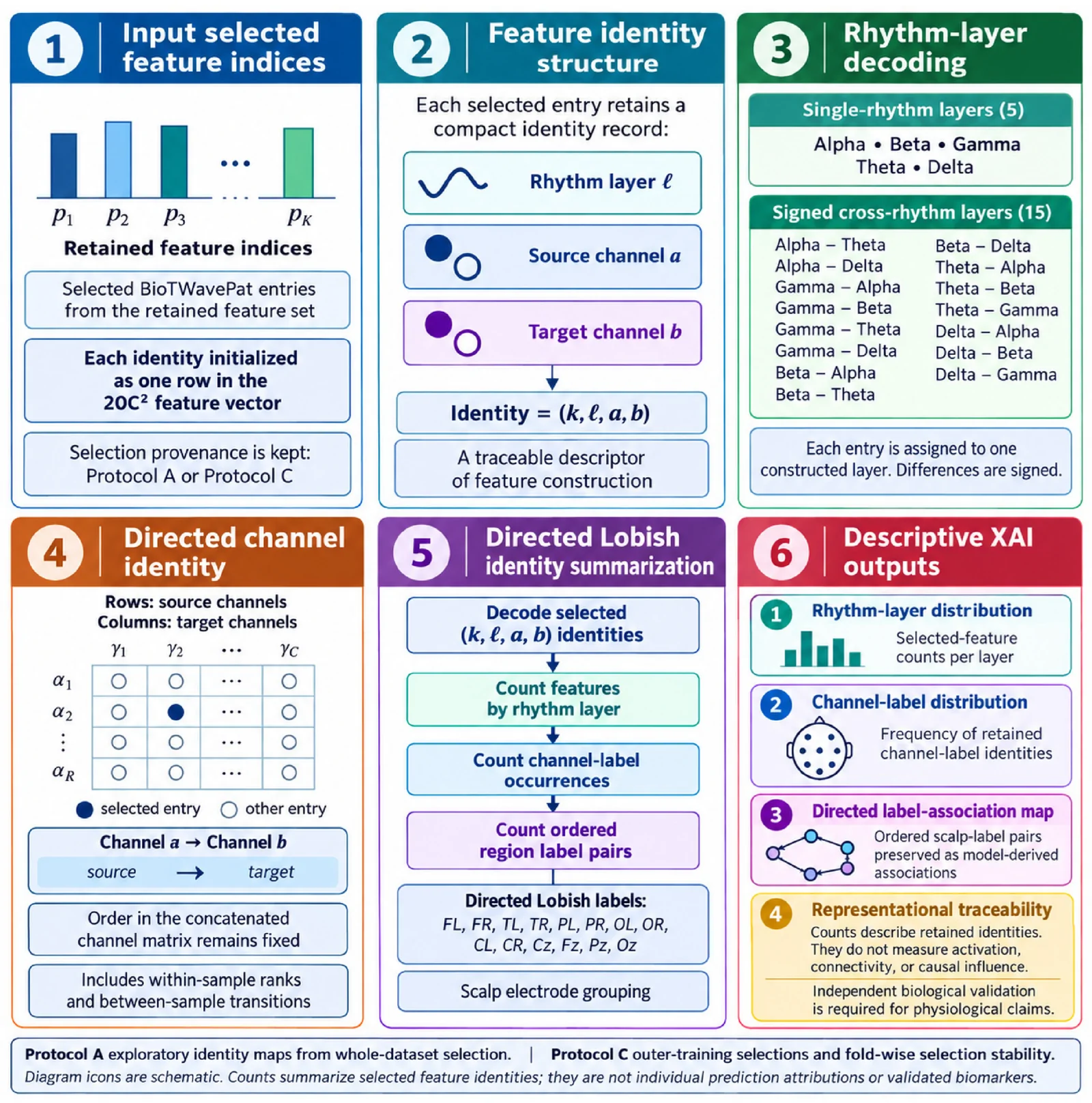
XAI generator. Schematic overview of the proposed XAI generation pipeline. Selected BioTWavePat features are decoded into feature identities, assigned to rhythm layers and directed channel relations, summarized through Directed Lobish labels, and converted into descriptive XAI outputs, including rhythm-layer distributions, channel-label distributions, directed label-association maps, and representational traceability summaries. The figure summarizes retained feature identities and does not imply individual prediction attribution or validated physiological interpretation.

To generate XAI results, EEG rhythms and Directed Lobish [19] were used together. The objective of this phase was to decode and summarize the identities of selected BioTWavePat features as model-derived directed feature associations. In BioTWavePat, each feature has a clear identity because each feature is generated from one rhythm layer and one directed channel transition. Therefore, the construction of retained features can be inspected, without treating their identities as quantified contributions to an individual prediction. The schematic illustration of this XAI generation model is demonstrated in Figure 5.

Figure 5 summarizes the decoding of selected feature identities into rhythm-layer and channel-label counts. These summaries describe the selected representation; they do not establish neurophysiological importance.

Directed Lobish uses 14 regional labels: FL, FR, TL, TR, PL, PR, OL, OR, CL, CR, Cz, Fz, Pz, and Oz. They identify left/right frontal, temporal, parietal, occipital and central electrode groups, together with central, frontal, parietal and occipital midline labels. Expressions such as FL → FR, TL → TR and Fz → Cz denote model-derived directed feature associations between these labels. The labels refer to scalp electrode groupings and do not localize cortical generators.

BioTWavePat generates 20 rhythm layers. These layers include five single-rhythm layers and 15 cross-rhythm layers. The single-rhythm layers are alpha, beta, gamma, theta, and delta. The cross-rhythm layers include alpha-beta, alpha-theta, alpha-delta, gamma-alpha, gamma-beta, gamma-theta, gamma-delta, theta-delta, theta-alpha, theta-beta, beta-alpha, beta-theta, beta-gamma, beta-delta, and alpha-gamma. Thus, each selected feature can be assigned to one constructed rhythm layer.

For each rhythm layer, the transition table uses row and column positions to identify the first and second channels in an adjacent pair of the concatenated ranked sequence. These positions define the source and target labels computationally. Each decoded feature therefore identifies a model-derived directed feature association:

(17) $$Channel_{i}\to Channel_{j}$$

Here, $Channel_{i}$: the source channel and $Channel_{j}$: the target channel.

After decoding, each selected entry is assigned its rhythm-layer, source-channel, and target-channel labels. The resulting symbolic statement identifies how the feature was constructed; it is not a physiological rule or a measured causal relation.

For example, a feature in the alpha-beta layer with source-channel label q and target-channel label w identifies the corresponding normalized transition-table entry. Selection of this entry shows that the feature was retained in the classification representation. It does not demonstrate an alpha-to-beta physiological influence or a neural transfer from channel q to channel w.

The Directed Lobish phase uses these decoded feature identities. First, the rhythm layer of each selected feature is found. Then, the source and target channels are found. After that, the selected features are counted according to rhythm layers and directed channel pairs. In this way, the model produces rhythm-level and channel-level XAI outputs.

Three descriptive outputs are generated: the distribution of selected features across rhythm layers, the frequency of channel-label occurrences, and a map of model-derived directed feature associations. Counts indicate how often feature identities occur in the selected set; they do not measure EEG activation, connection strength, or causal importance.

Direction arises from adjacency in the concatenated sequence of ranked channel indices in Equation (12). As specified in Equation (13), this sequence includes adjacent ranks within a time sample and pairs at the boundaries between successive samples. Thus, the arrow describes the order of two channel labels in a constructed sequence, rather than a measured physiological transmission. The normalized counts are descriptors of this sequence and are not estimators of directed information flow, functional or effective connectivity, or causal interaction. Directed Lobish supplies a traceable summary of selected feature identities; independent validation is required before neurophysiological or biomarker interpretations can be assigned.

Directed Lobish expresses selected feature identities in a readable symbolic form. For example, an alpha-beta entry identifies a constructed rhythm-difference layer and an ordered channel-label pair. This notation supports inspection of the representation without asserting biological interaction between the named rhythms or regions.

The XAI phase decodes rhythm-layer, source-channel, and target-channel identities from selected BioTWavePat entries. These identities provide descriptive model-derived directed feature associations. They support representation traceability without establishing biological mechanisms.

We performed feature-group ablations, participant-label permutations, fold-wise selection-stability analyses, and montage-opportunity comparisons (Appendix A.6). These experiments examine representation properties rather than validating physiological connectivity or individual prediction attribution. The appendix distinguishes Protocol A controls, Protocol C fold-wise stability, and the same-partition classifier benchmarks.

### 3.5. Computational Complexity and Scalability

The analysis separates representation construction, model selection, and fixed-model inference. Let C denote EEG channels, L samples per segment, N stored segments, and P outer held-out units (participants for STEW/MAT; recorded identifier groups for TMPD). The layer count is fixed at 20, and the stored preselection dimension is *d =* 20*C*^2^. Let *T_band(C, L)* denote the cost of producing the five filtered rhythm signals, including any associated filter setup. The filter algorithm, order, and boundary implementation are not specified sufficiently to assign a more detailed bound. Applying already estimated robust-normalization parameters and forming the signed difference maps require *O(CL)* and *O*(15*CL*) arithmetic operations, respectively. Estimating training-partition normalization statistics is a separate cost.

Under comparison-based channel sorting, Equation (12) requires *O*(20*LC log C*) operations. Equation (13) *counts* 20(*CL* − 1) adjacent pairs, while initializing, normalizing, and concatenating the tables requires *O*(20*C*^2^) operations. Thus, representation construction per segment is bounded by *O(T_band(C, L) +* 20*LC log C +* 20*C*^2^), excluding estimation of training-derived preprocessing parameters. A layer-at-a-time implementation requires *O(CL +* 20*C*^2^) storage for signal buffers, a ranked sequence, a transition table, and the output vector. Materializing all layers requires *O*(20*CL +* 20*C*^2^) storage. These bounds describe algorithmic storage.

A dense *N × d* feature matrix occupies *8Nd* bytes when stored as double-precision values, before array metadata, temporary copies, and selector workspaces. Appendix A.5 Table A9 illustrates this payload scaling. Retaining m selected features reduces the classifier input dimension to m but does not remove the cost of extracting d entries. For one query, exhaustive distance evaluation against *N_train* stored training segments costs *O(N_train m)* for a standard coordinate-wise distance. Neighbor retrieval adds an implementation-dependent cost, such as *O(N_train log N_train)* when all distances are sorted. Multiple retained tkNN models require summing their individual costs. Decoding and counting K retained feature occurrences requires *O(K)* operations, excluding initialization of summary arrays and figure rendering.

We fit the selectors once for Protocol A and once per outer training fold for Protocol C. Within each Protocol C training fold, we validate tkNN/IMV outcomes in the fixed selected feature space. We calculate the representation-construction and classifier costs separately and report dense feature-array storage in Appendix A.6 Table A17.

## 4. Experimental Results

We compare the original analysis with training-fold outcome selection and the fully fold-internal pipeline. Table 4 and Table 5 report distinct segment and aggregated decision endpoints. Protocol C is the primary participant-independent estimate.

### 4.1. Experimental Setup

The proposed model was implemented as an explainable feature engineering pipeline. First, BioTWavePat was used to extract rhythm-based directed transition features. Then, cumulative weighted feature selection was applied. Four selectors were used: CWNCA, CWChi2, CWmRMR, and CWReliefF. Binary intersections were also generated. Thus, ten selected feature vectors were obtained.

We evaluated three protocols: Protocol A—original analysis; Protocol B—training-fold outcome selection; and Protocol C—fully fold-internal pipeline (leakage-free). Protocol A fits normalization and feature selection once on all participants and chooses the final candidate by pooled LOSO accuracy. Protocol B restricts outcome choice to the training fold but retains Protocol A feature selection. Protocol C re-estimates all data-dependent steps in each outer training fold; it supplies the unbiased participant-independent estimates under this design. The outer held-out unit was one participant for STEW and MAT and one recorded identifier group for TMPD. TMPD contained 55 participants under 54 identifiers: two same-class participants shared one identifier and were withheld together in the same fold. All 55 participants were included; no participant was excluded. Thus, TMPD used 54 identifier-group-held-out folds rather than 55 one-person LOSO folds. In Protocol C, normalization, selectors, and thresholds were fitted only on the outer training data. Candidate tkNN/IMV outcomes were compared by inner validation in the fixed outer-training feature space. Outer-test predictions or labels were not used for outcome choice. This design is fully internal to the outer training partition without fully nested feature-selection refits.

Performance metrics were computed from pooled outer-test predictions at segment and aggregated-decision levels. Class 0 denotes the dataset-specific low category and Class 1 the high category. In STEW, these categories refer to participant self-reported workload; they are not assumed to represent the same construct as arithmetic performance. With Class 1 positive, *sensitivity = TP/(TP + FN), specificity = TN/(TN + FP)*, and balanced accuracy is their mean. Macro-F1 is the unweighted mean of the class-specific F1 scores. Positive-class F1 is retained separately in Appendix A.2 Table A5 and Table A6.

We calculated descriptive 95% Wilson accuracy intervals for the aggregated decisions in Table 5. Appendix A.4 Table A8 reports class-recall intervals for Protocols A and C; Protocol A performance estimates are subject to whole-dataset selection bias. Appendix A.6 Table A13 includes naive segment Wilson intervals, which assume independent segments and therefore understate dependence within participants. We also report a participant-cluster interval based on a beta model of within-participant accuracy for the original analysis. These intervals do not account fully for overlapping LOSO training sets or refitting uncertainty.

We summarized held-out-unit segment accuracy by mean and standard deviation and performed exploratory McNemar and Wilcoxon comparisons. STEW/MAT units were participants; TMPD units were recorded identifier groups. Wilcoxon versus EEGNet used Protocol A. McNemar comparisons used original Protocol A component predictions; the continuity-corrected statistic and separate discordant counts are given in Appendix A.6 Table A19. These exploratory tests do not establish a Protocol C improvement.

### 4.2. Classification Results

Table 4 compares segment-level accuracy, balanced accuracy, macro-F1, class recalls, and MCC across Protocols A, B, and C. Protocols B and C correspond to outcome-only and complete training-fold reruns, respectively. Appendix A.2 Table A5 retains original positive-class metrics, and Table A18 gives additional metrics.

**Figure 6:**
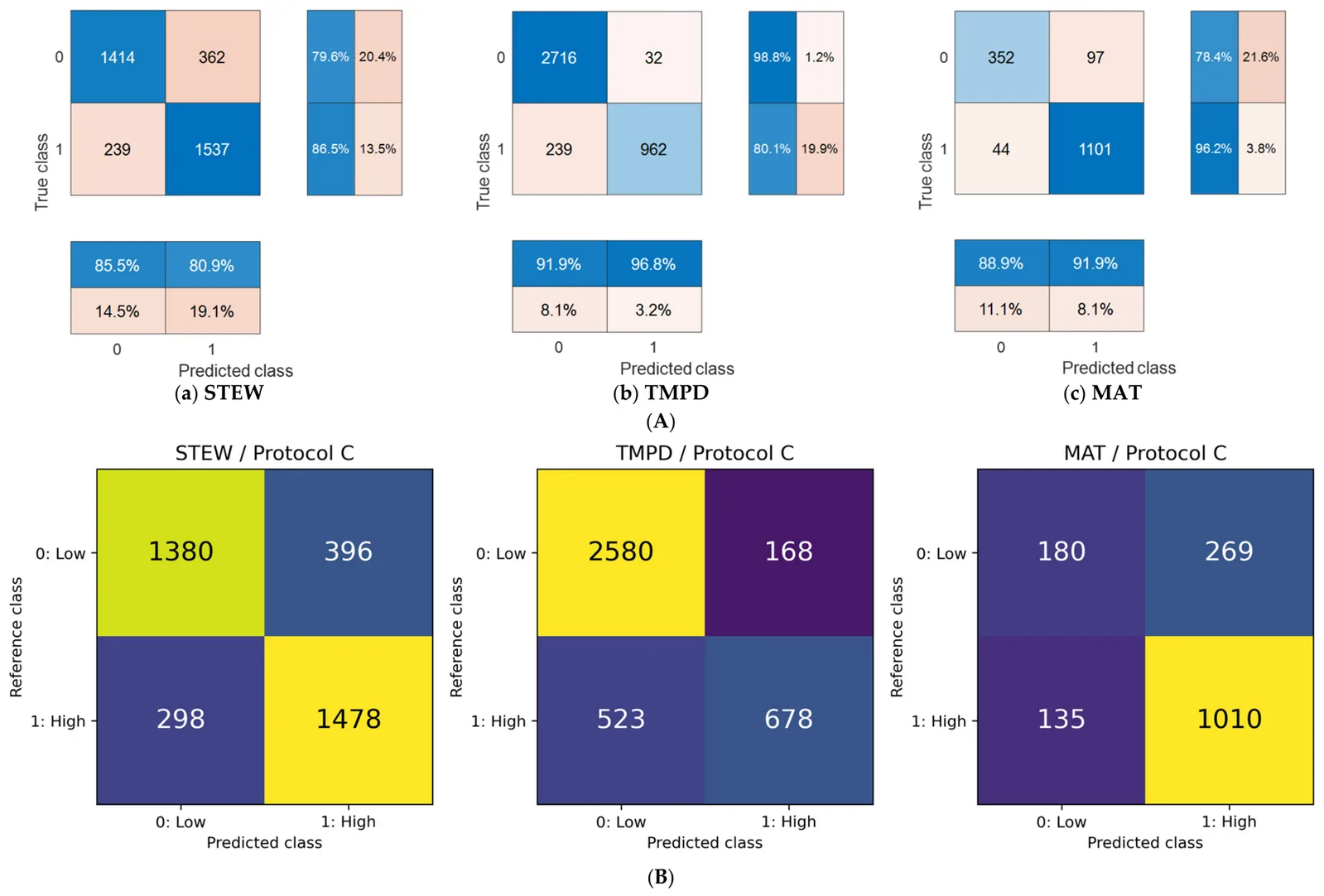
(**A**). Protocol A segment confusion matrices (0: low; 1: high). (**B**). Protocol C segment confusion matrices: STEW (TN 1380, FP 396, FN 298, TP 1478), TMPD (2580, 168, 523, 678), MAT (180, 269, 135, 1010). Class 0: low; Class 1: high.

Table 4 shows that restricting outcome choice alone changes accuracy by 0.73, 0.61, and 0.37 percentage points for STEW, TMPD, and MAT, respectively. Re-estimating the entire pipeline has a substantially larger effect. Figure 6A retains Protocol A matrices, and Figure 6B shows Protocol C matrices.

Figure 6A retains the original correct segment counts: 1414/1537 (STEW Classes 0/1), 2716/962 (TMPD), and 352/1101 (MAT). These counts describe the original Protocol A analysis and are subject to whole-dataset selection bias.

Figure 6B presents the Protocol C segment confusion matrices. Table A20 records the Protocol B counts for completeness. Figure 6B shows the low Class 1 sensitivity in TMPD and low Class 0 specificity in MAT. These fully fold-internal results support a substantially more cautious interpretation than the original analysis.

We distinguish pooled segment accuracy, variability in each participant’s segment accuracy, and accuracy of aggregated decisions. Appendix A.6 Table A13 gives participant-wise segment summaries; Table 5 gives the aggregation endpoint.

Protocol A to Protocol C segment accuracy decreases were 2.62 percentage points for STEW, 10.64 for TMPD, and 16.50 for MAT; balanced accuracy decreased by 2.62, 14.30, and 23.13 points. The small outcome-only changes in Protocol B identify whole-dataset feature selection as the main source of optimism. Under Protocol C, MAT accuracy was 74.65%, close to its 71.83% majority-class reference, with only 40.09% Class 0 specificity. TMPD Class 1 sensitivity was 56.45%. These class errors preclude a claim of meaningful MAT discrimination under Protocol C.

Table 5 reports correct/total aggregated decisions and descriptive 95% Wilson intervals across the three protocols, with class-aware metrics for Protocols A and C. STEW and MAT use one decision per participant; TMPD uses one decision per recorded identifier group. All segments vote equally, and an exact tie is assigned to Class 1. Protocol A additional class-aware metrics are retained in Appendix A.6 Table A18.

Under Protocol C, STEW correctly classified 22/24 low-performance and 21/24 high-performance participants (balanced accuracy 89.58%), TMPD 35/37 low-performance and 9/17 high-performance identifier groups (balanced accuracy 73.77%), and MAT 6/10 low-performance and 21/26 high-performance participants (balanced accuracy 70.38%). The low TMPD high-performance sensitivity (52.94%) and MAT low-performance specificity (60.00%) mirror the class errors observed at segment level.

Each segment contributed one vote to its participant decision in STEW/MAT or identifier-group decision in TMPD. An exact vote tie was assigned to Class 1 (high performance). The two TMPD participants sharing an identifier belonged to the same reference class, so their group had one unambiguous reference label. Aggregated and segment accuracies use different units and denominators.

All 55 TMPD participants were included. Two same-class participants shared one recorded identifier, giving 54 held-out identifier groups (37 low, 17 high). Their segments were kept together in one outer test fold. Protocol A correctly classified 51/54 groups (94.44%), and Protocol C correctly classified 44/54 groups (81.48%). These are identifier-group results, not 54-person results or a reanalysis using 55 separate participant folds.

STEW used participant-level self-report labels (24 low, 24 high), with 74 segments per participant. Protocol A correctly classified 45/48 participants: 93.75% (Wilson 83.16–97.85%). Protocol C correctly classified 43/48: 89.58% (77.83–95.47%).

TMPD Protocol A identifier-group accuracy was 94.44% (51/54; Wilson 84.89–98.09%), with group Class 1 sensitivity 82.35% and Class 0 specificity 100.00%. Protocol C group accuracy was 81.48% (44/54; Wilson 69.16–89.62%). The majority-class group reference was 37/54 = 68.52%.

MAT Protocol A aggregated accuracy was 94.44% (34/36; Wilson 81.86–98.46%), with Class 1 sensitivity 100.00% and Class 0 specificity 80.00%. Protocol C accuracy was 75.00% (27/36; Wilson 58.93–86.25%). Predicting the majority class correctly classifies 26/36 = 72.22%, so the Protocol C count is only one correct participant higher.

**Figure 7:**
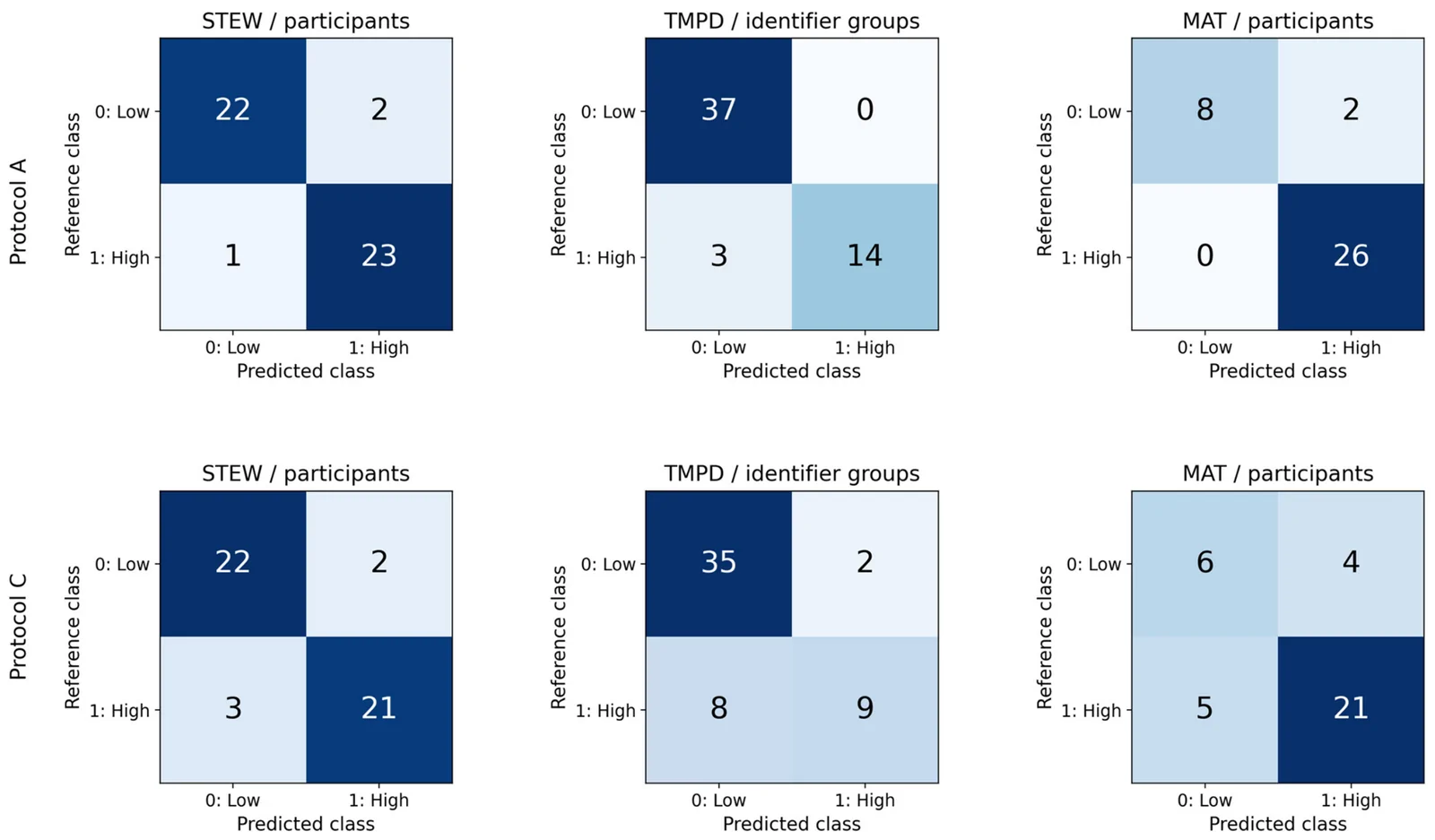
Aggregated confusion matrices under Protocol A (**top**) and Protocol C (**bottom**). STEW/MAT decisions are participant-level; TMPD decisions are identifier-group-level (54 groups containing all 55 participants).

Appendix A.4 Table A8 reports class-recall intervals under Protocols A and C for STEW/MAT participants and TMPD identifier groups, corresponding to Figure 7. Small class denominators produce broad uncertainty.

### 4.3. Component-Level Performance Analysis

Table 6 reports Protocol A selector, intersection, and IMV component outcomes. The final candidate was chosen by the maximum pooled LOSO accuracy; this is distinct from training-fold outcome choice in Protocols B and C.

Table 6 shows different Protocol A single-selector maxima across datasets: CWChi2 for STEW and CWmRMR for TMPD and MAT. Such rankings cannot establish a preferred selector under Protocol C. Protocol A intersections retain entries shared by whole-dataset selector fits. Appendix A.1 Table A1, Table A2, Table A3 and Table A4 retain these component results and selected-feature counts; their estimates are subject to whole-dataset selection bias. They do not measure fold-internal selection stability.

Protocol A best individual versus IMV segment accuracies were 80.94% versus 83.08% (STEW), 91.24% versus 93.14% (TMPD), and 91.15% versus 90.65% (MAT). The pooled-accuracy maximum defined the original final outcome. Appendix A.6 Table A19 reports exploratory paired comparisons using the confirmed discordant counts.

### 4.4. Explainable Results

**Figure 8:**
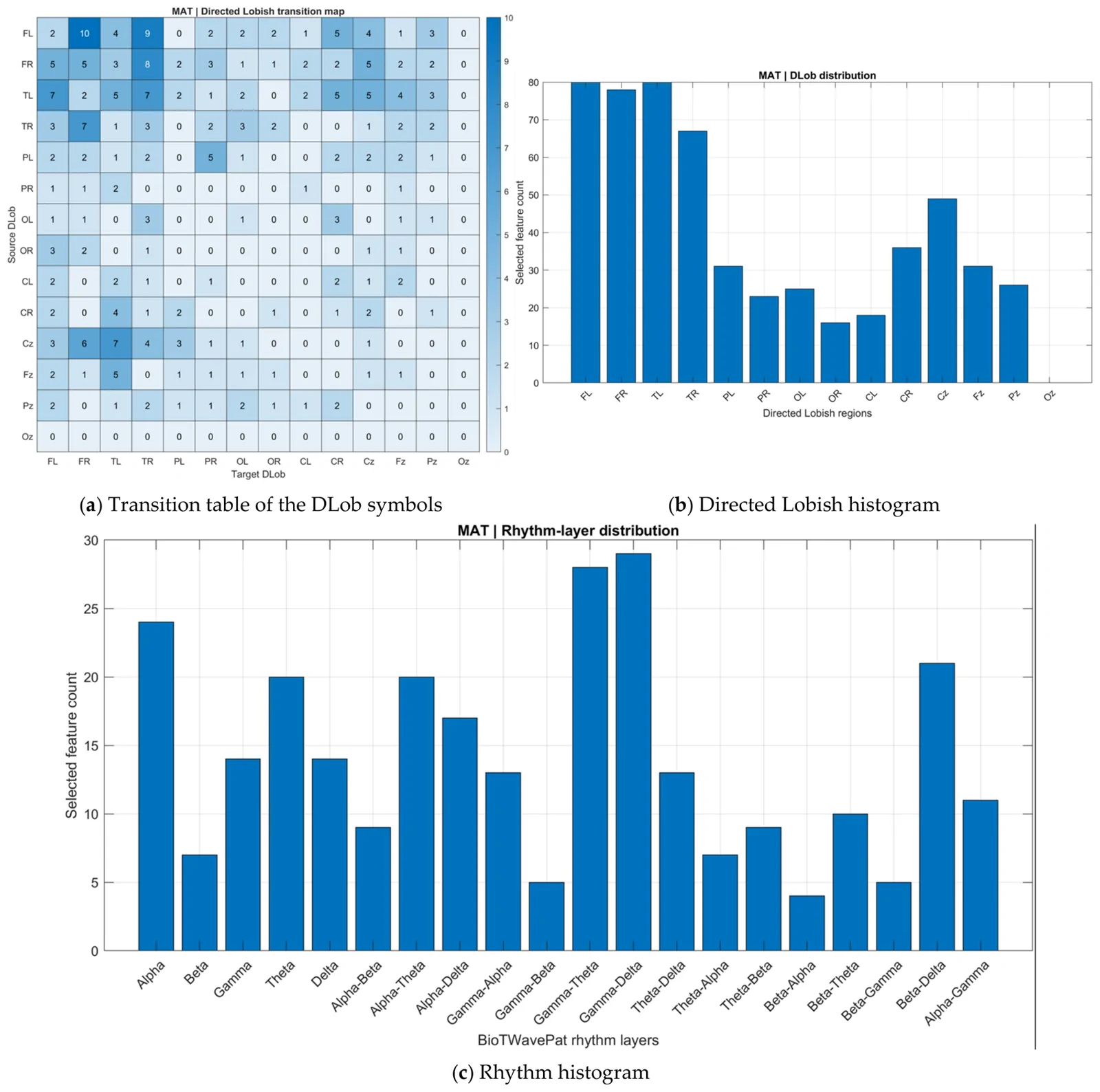
XAI results for MAT dataset. Derived from whole-dataset selection (Protocol A).

**Figure 9:**
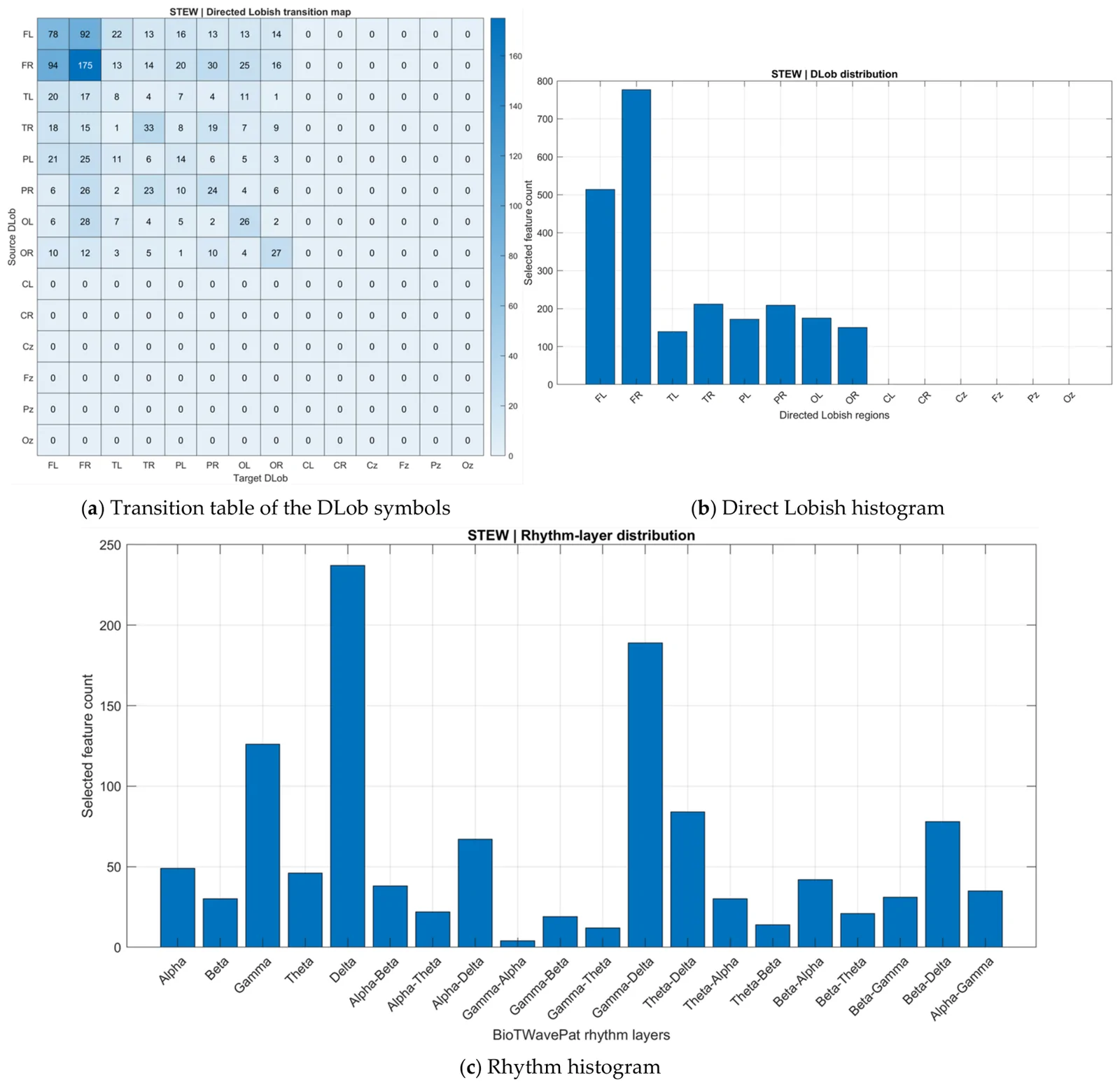
XAI results for STEW dataset. Derived from whole-dataset selection (Protocol A).

**Figure 10:**
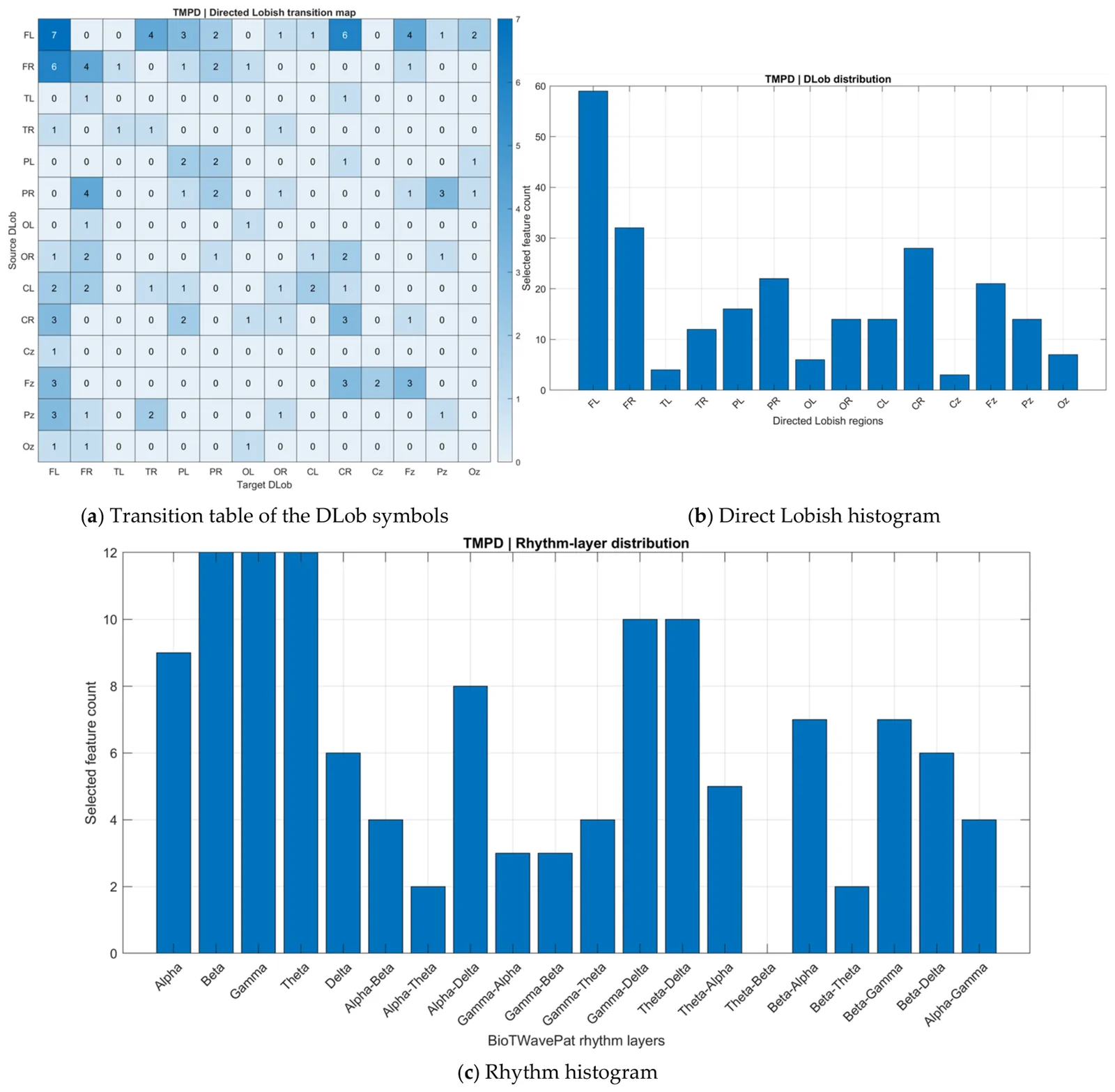
XAI results for TMPD dataset. Derived from whole-dataset selection (Protocol A).

**Figure 11:**
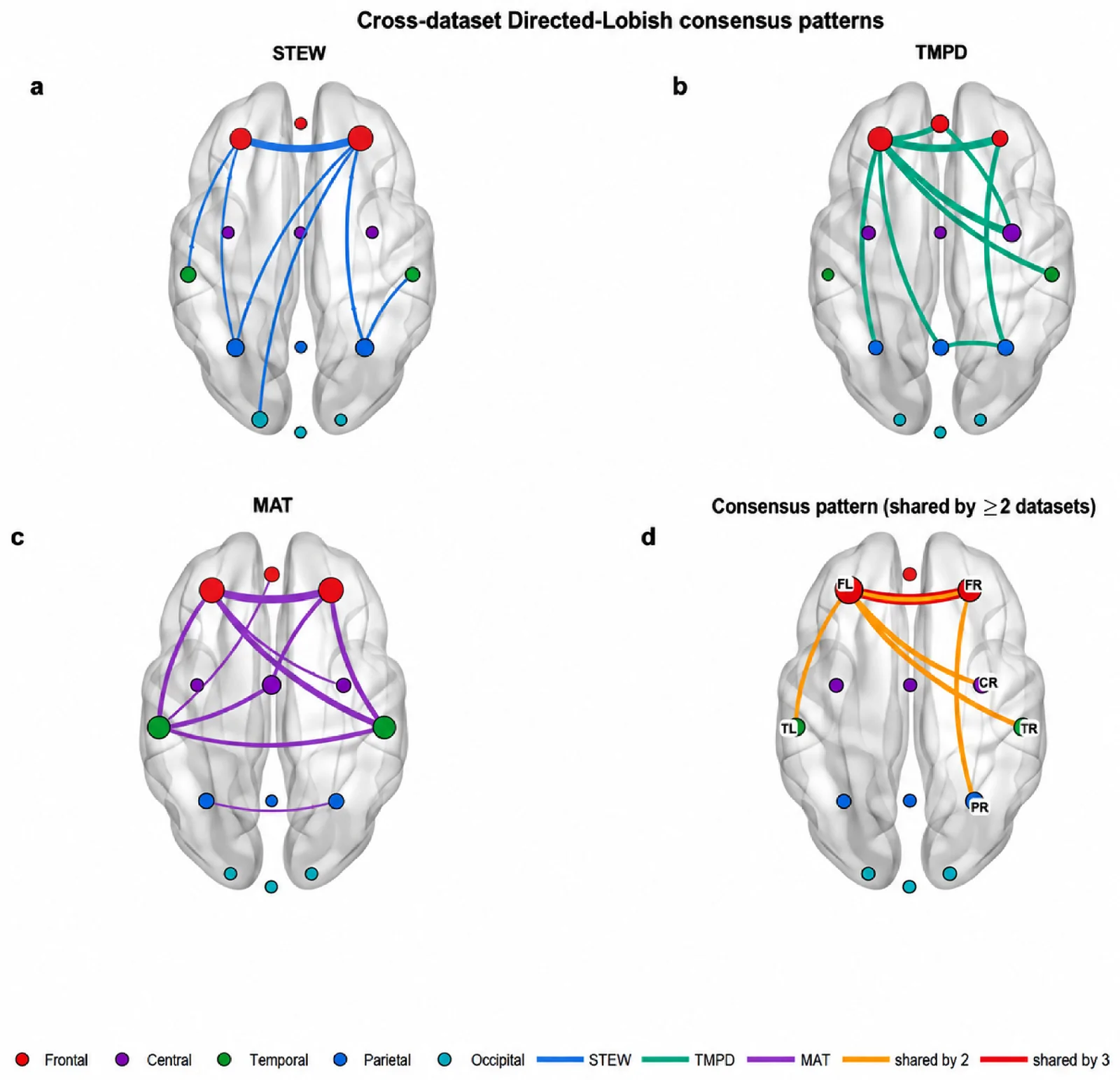
Recurrence of selected feature-label associations across three separately analyzed datasets ((**a**) STEW, (**b**) TMPD, (**c**) MAT (**d**) Consensus pattern). Shared labels identify descriptive overlap; channel coverage, spectral support, and prediction endpoints were not harmonized to establish common neural mechanisms. Derived from whole-dataset selection (Protocol A).

Table 7 and Figure 8, Figure 9, Figure 10 and Figure 11 are derived from whole-dataset selection (Protocol A), including the MAT CWmRMR set of 280 features. Weighted counts include repeated retained identities, whereas unique counts denote distinct identities. These post hoc summaries describe selected feature composition; they are not Protocol C explanations or tests of biological relevance.

Table 7 shows dataset-specific distributions of selected feature identities. STEW had frequent delta-related entries and frontal-label associations. TMPD had equal leading counts for beta, gamma and theta single-rhythm layers. MAT had frequent gamma-related cross-rhythm entries and frontal-temporal label associations. These distributions describe the composition of the selected representation; the counts do not establish physiological rhythm coupling or the strength of brain-region interactions.

Figure 8a summarizes model-derived directed feature associations after channel labels were grouped into Directed Lobish regions. Rows and columns retain the computational source and target labels. Higher values indicate more selected feature occurrences for a label pair. Frontal, temporal, central, and parietal labels occurred frequently. Supplementary montage-opportunity null comparisons are now reported in Appendix A.6 Table A16; they do not establish neural activation or effective connectivity.

FL, FR, TL, TR, Cz, and CR appeared in many source or target positions in Figure 8a. These occurrences locate selected feature identities within the scalp-label mapping. They do not show neuronal activation, cortical communication, or an attention or memory mechanism.

Figure 8b counts occurrences of electrode-group labels in the MAT selected-feature set. FL, TL, and FR had the highest counts, with TR, CR, and Cz also represented. These observations describe the labels attached to retained entries in Figure 8a. They do not rank cortical regions by activation, physiological importance, or contribution to a prediction.

Figure 8c summarizes the MAT rhythm-layer labels attached to selected entries. gamma-delta and gamma-theta had the highest counts; alpha, beta-delta, theta, and alpha-theta were also represented. These frequencies describe the retained representation. They do not establish that the cross-rhythm layers improved accuracy or measured physiological rhythm interactions.

The histogram in Figure 8c shows that several cross-rhythm layers occurred more frequently in the selected set than some single-rhythm layers. gamma-related and beta-related entries were prominent. This observation concerns constructed feature identities and does not demonstrate physiological cross-frequency coupling.

Figure 9a shows that FL → FR, FR → FL, and FR → FR had the highest selected-feature counts. These entries identify frequent model-derived directed feature associations involving frontal electrode labels in STEW. Their values are not measures of neuronal activity or connection strength.

Temporal, parietal, and occipital label pairs also occurred in the STEW selected-feature map, with fewer occurrences than frontal labels. Central and midline labels had no selected occurrences in this summary. Label coverage is constrained by the recorded montage, and zero counts do not demonstrate physiological inactivity. The map describes retained label pairs rather than a pattern of regional brain engagement.

Figure 9b shows that FR had the highest label-occurrence count in the STEW selected set, followed by FL. TR, PR, OL, PL, and OR also occurred. These counts describe which scalp-label groups were represented in the selected feature identities; they do not demonstrate frontal cortical dominance or quantify frontal contribution to classification.

Figure 9c shows the STEW rhythm-layer counts. delta was the most frequent single-rhythm label, and gamma-delta was the most frequent cross-rhythm label. gamma, theta-delta, beta-delta, and alpha-delta were also represented. The observation is restricted to selected feature identities and does not establish a delta-mediated physiological mechanism.

Figure 9c also shows frequent gamma-delta, theta-delta, beta-delta, and alpha-delta feature identities. These layer counts describe the selected representation. They do not establish interactions between oscillatory generators or the neurophysiological importance of delta activity.

Figure 10a shows the highest selected-feature counts around the FL label, including FL → FL, FL → TR, FL → CR, and FL → Fz. FR-based associations also occurred, especially FR → FL and FR → FR. These are model-derived directed feature associations between electrode-group labels, rather than measured neural activity or information flow.

The TMPD selected-feature map also contained CR, PR, PL, OR, Fz, and Pz labels, with fewer occurrences than frontal labels. These are differences in label frequency within the retained set. They do not show weaker physiological contributions from the corresponding regions, and the map is not a validated neural network.

Figure 10b shows that FL had the highest label-occurrence count in TMPD, followed by FR. CR, PR, Fz, PL, OR, CL, and Pz were also represented. Their inclusion identifies retained scalp-label groups; it does not establish activation of those regions or their contribution to individual decisions.

Figure 10c summarizes the TMPD rhythm-layer labels. beta, gamma, and theta were the most frequent single-rhythm labels, and alpha was also represented. gamma-delta and theta-delta had frequent cross-rhythm entries; alpha-delta, beta-alpha, beta-gamma, and beta-delta also occurred in the selected set. These observations do not quantify each layer’s predictive contribution or physiological relevance.

Figure 10c shows selected identities from several single-rhythm and cross-rhythm layers. beta, gamma and theta single-rhythm entries were frequent, together with gamma-delta and theta-delta entries. These distributions describe representation composition and do not demonstrate physiological rhythm coordination.

## 5. Discussion

BioTWavePat-XFE combines rhythm-channel transition features, four cumulative-weighted selectors, tkNN/IMV classification, and Directed Lobish decoding. The main finding is the effect of whole-dataset selection on performance, alongside traceable feature identities.

Protocol A segment accuracies were 83.08%, 93.14%, and 91.15% for STEW, TMPD, and MAT; they are subject to whole-dataset selection bias. Fully fold-internal Protocol C accuracies were 80.46%, 82.50%, and 74.65%, with balanced accuracies of 80.46%, 75.17%, and 64.15%. The reduction is an empirical finding about selection leakage, especially for TMPD and MAT. Participant-independent estimation requires exclusion from every data-dependent stage, not merely the classifier split.

Protocol A aggregated accuracies were 93.75% (STEW participants), 94.44% (TMPD identifier groups), and 94.44% (MAT participants). Protocol C obtained 89.58%, 81.48%, and 75.00%, respectively. TMPD includes all 55 participants, but its aggregation denominator is 54 recorded groups. MAT Protocol C correctly classified 27/36 participants, versus 26/36 for majority prediction.

Original-analysis held-out-unit segment accuracy had mean/SD 83.08%/17.2 points for STEW participants, 93%/18.5 points for TMPD identifier groups, and 91.2%/18 points for MAT participants (Appendix A.6 Table A13). TMPD and MAT medians were 100% and 97.7%; the STEW median is not reported. An aggregated correct decision does not imply uniformly high segment accuracy within each unit.

Protocol C reveals class-specific weaknesses: TMPD high-performance sensitivity was 56.45%, and MAT low-performance specificity was 40.09%. MAT accuracy of 74.65% was close to its 71.83% majority-class segment reference, and its aggregated result exceeded majority prediction by only one correct participant. We therefore do not claim meaningful MAT discrimination. Class-aware metrics and uncertainty are essential to interpreting the result.

The descriptive XAI summaries showed frequent FL and FR electrode-group labels across the three datasets. Temporal labels also appeared frequently in MAT, with central and parietal labels represented in MAT and TMPD. These observations concern selected feature identities. They do not establish the activation or causal role of the corresponding cortical regions.

The rhythm-layer summaries included both single-rhythm and cross-rhythm feature identities. delta-related, gamma-related, beta-related and theta-related entries occurred in the selected sets. This supports the traceability of the representation across its constructed layers. It does not independently establish physiological coupling between EEG rhythms.

The three datasets had different selected-feature profiles. MAT included frontal, temporal, central, and parietal labels, with frequent gamma-delta and gamma-theta entries. STEW included frequent frontal-label associations and Delta-related entries. TMPD included frequent frontal labels and beta, gamma, theta, gamma-delta and theta-delta entries. Figure 11 summarizes shared model-derived directed feature associations; recurrence across datasets is a descriptive observation rather than independent neurophysiological validation.

Figure 11 shows dataset-specific feature-label maps in panels (a)–(c) and associations shared by at least two datasets in panel (d). FR→FL appeared in all three datasets. FL → TR, FL → FR, FL → CR, PR → FR, and TL → FL also appeared in the shared set. The arrows retain the order of labels in the feature construction and do not represent cortical information flow, connectivity, or causality.

Table 8 lists the shared model-derived directed feature associations. FR → FL appeared in all three datasets, and the other listed associations link frontal labels with temporal, central, or parietal labels within the representation. These observations describe recurrent feature identities. They do not demonstrate communication between the underlying brain regions.

Table 8 summarizes recurrence after dataset-specific channel labels were mapped to common region names. A shared name can represent different electrode subsets and different numbers of available channel pairs. Accordingly, label overlap is a descriptive indexing result; it does not establish equivalent anatomical sampling, statistically enriched recurrence, or a shared biological mechanism. Section Dataset Heterogeneity and Interpretation Scope explains these acquisition and endpoint limitations.

### 5.1. Comparisons

Table 9 stratifies published results by validation protocol and prediction unit. Protocol A and Protocol C occupy separate rows for this study. Protocol C excludes the full held-out unit from every data-dependent step. TMPD rows identify its 54 recorded groups rather than 55 one-person folds. Literature values differ in cohorts, tasks, preprocessing, and aggregation; they provide context rather than a controlled performance ranking. Appendix A.6 Table A14 and Table A15 separately report our classifier and Protocol A extractor experiments.

Table 9 separates the validation design from the unit used to calculate accuracy. LOSO holds out a participant, but a reported LOSO accuracy can summarize segment predictions, fold averages, or an aggregated participant decision. Participant-grouped 10-fold evaluation also separates participants, whereas sample-wise 10-fold evaluation does not impose this restriction. Other k-fold and split-based results are not assumed to be participant-independent when that information is unclear. The MAT comparison result reported for MountPat is retained in a separate unresolved group because its evaluation section and comparative discussion give conflicting accounts of LOSO availability. This result is not treated as a confirmed matched-protocol benchmark.

TMPD Protocol A segment/group accuracies were 93.14%/94.44%; Protocol C obtained 82.50%/81.48% under the same 54 identifier-group partitions covering all 55 participants. Appendix A.6 Table A14 compares classifiers using those same outer partitions and training-only tuning. Appendix A.6 Table A15 uses Protocol A for earlier-extractor comparisons and therefore does not establish an advantage under Protocol C.

### 5.2. Interpretation of Model-Derived Feature Associations

**Figure 12:**
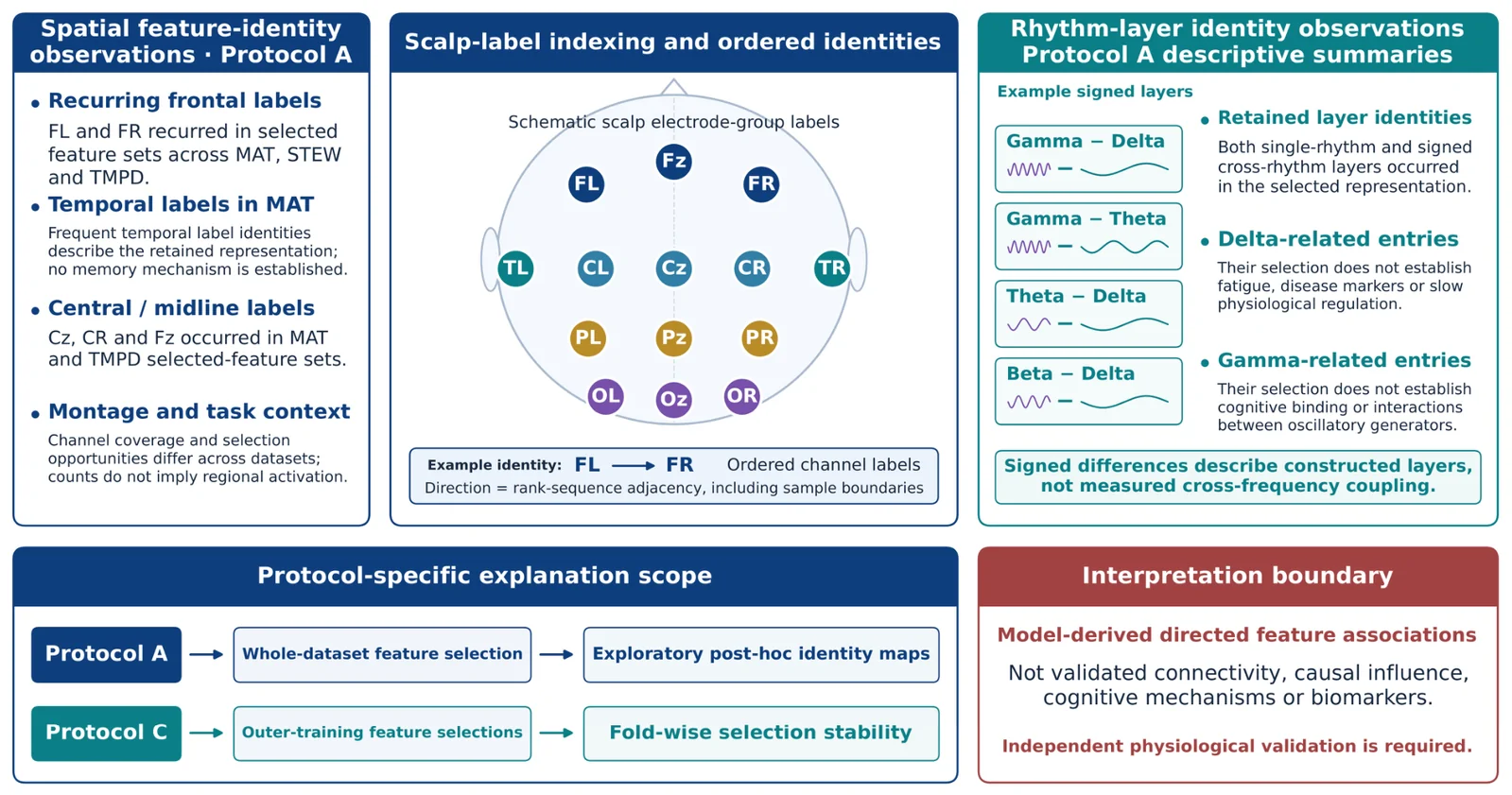
Interpretation boundaries for model-derived directed feature associations.

Figure 12 summarizes the interpretation boundaries of the selected feature identities. The diagram separates descriptive observations from physiological conclusions that were not established by the present analysis.

Figure 12 identifies frequent frontal electrode-group labels as a descriptive property of the selected feature sets. FL and FR recurred across MAT, STEW, and TMPD. Scalp-region labels provide a spatial indexing convention; they do not resolve cortical sources. Accordingly, frontal-label recurrence cannot establish executive control, working memory, or another specific cognitive mechanism.

An arrow between two regional labels represents their ordered occurrence in the constructed channel-index sequence. The sequence combines within-sample ranks with boundaries between successive samples. Consequently, these arrows cannot be interpreted as the direction of cortical transmission, top-down control, or effective connectivity.

Temporal electrode-group labels occurred frequently in MAT. Their occurrence may motivate independent, task-specific investigations but does not demonstrate memory-related or associative processing. Differences between the dataset maps remain descriptive and should be considered alongside task and montage differences.

Cz, CR, and Fz were represented in the MAT and TMPD selected-feature sets. These labels identify scalp recording locations or groups. Their counts do not establish sensorimotor integration, attention regulation, or midline cognitive control.

Both single-rhythm and cross-rhythm layers occurred in the selected representation. Cross-rhythm layers are computational constructions specified by the method. Their selection does not demonstrate cross-frequency coupling, oscillatory coordination, or communication between neural generators.

delta-related entries are model-derived features. Their occurrence does not establish a disease marker, fatigue mechanism, or slow regulatory process. Such hypotheses require independent analyses with appropriate artifact control and physiological measurements.

gamma-delta and gamma-theta entries likewise describe constructed layer identities. Their recurrence does not demonstrate cognitive binding or interactions between fast and slow oscillatory generators. Interpretation should remain at the feature-association level until independently validated.

Directed Lobish therefore provides an auditable summary of selected feature identities. The present findings are model-derived directed feature associations, not validated information-flow measures, connectivity estimates, causal interactions, or biomarkers. Recurrence across the three datasets does not remove this interpretation boundary.

#### Dataset Heterogeneity and Interpretation Scope

Table 2 documents substantial acquisition differences: STEW uses 14 channels at 128 Hz with Emotiv EPOC, TMPD uses 32 channels at 256 Hz with Emotiv Flex 2 Saline, and MAT uses Neurocom recordings at 500 Hz. Channel count changes the 20C^2^ feature universe and the number of channel-pair identities available for selection. Mapping channels to the same Directed Lobish region name does not make electrode coverage identical. Regions containing more recorded channels can supply more candidate identities; raw selected-feature counts do not correct for this opportunity difference. Neither common region labels nor similar counts demonstrate anatomical equivalence.

Sampling frequency changes the number of samples in a fixed-duration segment and the temporal spacing of the ranked observations. The constructed sequence includes both within-sample rank adjacency and boundaries between samples; changes in montage and sampling can therefore alter its descriptors. Acquisition hardware and preprocessing may also affect signal quality and spectral content. The sampling rates impose different Nyquist limits, so a gamma band specified only as above 30 Hz need not have identical upper-frequency support across datasets. A common band name is insufficient to establish identical filtering or comparable oscillatory content. These factors were not isolated in a controlled device or montage experiment.

The prediction endpoints also differ. STEW uses a multitasking workload setting, MAT uses arithmetic performance, and TMPD uses a mental-test-score threshold. A low/high class name does not establish that these endpoints measure one latent cognitive trait. Device, montage, sampling, and task are tied to dataset identity in this evaluation; their individual effects cannot be separated from the reported summaries. Models were fitted separately within each dataset, and no frozen cross-dataset transfer or leave-one-dataset-out result is reported. Recurring frontal-label or rhythm-layer identities therefore remain descriptive replication targets. They do not establish common neural mechanisms, a device-invariant biomarker, or a unified physiological signature.

### 5.3. Findings and Advantages

#### 5.3.1. Findings

Protocol C produced segment accuracies of 80.46% (STEW), 82.50% (TMPD), and 74.65% (MAT), compared with Protocol A values of 83.08%, 93.14%, and 91.15%. Accuracy decreases of 2.62, 10.64, and 16.50 points and balanced-accuracy decreases of 2.62, 14.30, and 23.13 points identify selection leakage as a main finding. Separate within-dataset reruns do not demonstrate cross-dataset transfer.

Protocol C correctly classified 43/48 STEW participants, 44/54 TMPD identifier groups, and 27/36 MAT participants, giving 89.58%, 81.48%, and 75.00%, respectively. All 55 TMPD participants contributed segments, with two same-class participants combined in one held-out group. MAT majority prediction gives 26/36 correct decisions. These results do not demonstrate a substantial MAT advantage over majority prediction.

Frontal labels occurred frequently in the Protocol A selected-feature summaries. The montage-opportunity comparisons in Appendix A.6 Table A16 did not show frontal-label enrichment in STEW, TMPD, or MAT (approximate one-sided *p* = 0.9835, 0.0565, and 0.9617). The frontal opportunity fractions use eight frontal electrodes among 14 in STEW, 10 among 32 in TMPD, and the standard 19-channel MAT montage. Selected features are dependent; these unadjusted tests describe montage coding rather than physiology.

The selected representation contained frontal-label associations and associations involving temporal, central, and parietal labels. Their distributions differed across MAT, STEW, and TMPD. These model-derived directed feature associations describe the representation; they do not show regional information flow.

Single-rhythm and cross-rhythm identities were selected across the datasets. delta-related, gamma-related, theta-related, and beta-related entries occurred in different profiles. These counts show which constructed layers were represented, without establishing physiological rhythm coordination.

Table 6 reports original selector and voting rankings under Protocol A. Protocol B isolates outcome choice, whereas Protocol C re-estimates feature selection as well. The larger Protocol C reductions show why pooled-test maxima should not determine participant-independent performance claims.

#### 5.3.2. Advantages

- Each retained feature has a rhythm-layer identity and an ordered channel-label pair. These identities permit the construction of selected entries to be inspected and expressed symbolically. This provides representation traceability; it does not establish a quantitatively validated attribution of individual predictions.
- BioTWavePat retains single-rhythm and signed rhythm-difference layer identities. alpha-beta and beta-alpha are distinct stored labels with algebraically related maps. Their inclusion broadens the indexed representation, without establishing independent physiological interactions, information gain, or an accuracy benefit from those layers.
- The transition tables retain the order of channel labels in the ranked sequence. This permits each selected entry to be traced to its computational source and target labels. The retained direction is a representation property, rather than a neural transmission estimate.
- CWNCA, CWChi2, CWmRMR, and CWReliefF were used together. In addition, binary intersections were generated. This approach creates different selected feature spaces. Therefore, the classifier can use both selector-specific and intersection-based information.
- Protocols B and C select tkNN/IMV outcomes by inner validation using only outer-training data and a fixed selected feature space. Protocol C additionally fits normalization and feature selection within each outer fold. The full held-out participant or TMPD identifier group remains excluded from both stages.
- Protocol C maintains participant separation at every data-dependent stage. The four selectors are fitted once per outer training fold, and their feature indices are kept fixed during inner outcome validation. TMPD uses the recorded identifier groups, including one group with two same-class participants.
- Directed Lobish and rhythm-layer analysis make selected feature identities readable at the channel-label and layer levels. This provides model transparency. Independent validation is required before these descriptive associations can support neurophysiological or biomarker claims.

### 5.4. Limitations and Future Work

#### 5.4.1. Limitations

Original estimates used whole-dataset feature selection; leakage-free estimates are lower. The Protocol C decline is particularly large for TMPD and MAT and changes the substantive interpretation of MAT discrimination.

- Acquisition hardware, electrode coverage, channel count, sampling frequency, task, and class definition vary together across the datasets. Their individual contributions cannot be identified from the present summaries. Coarse region mapping does not harmonize anatomical sampling, and raw feature counts do not adjust for available channel-pair opportunities. Separate within-dataset LOSO evaluation does not establish device-independent transfer. Consequently, cross-dataset label recurrence cannot support common neural mechanisms or a unified biomarker interpretation.
- The regional labels denote scalp electrode groupings and do not localize cortical generators. Ordered channel-index transitions are not measures of directed information flow, functional or effective connectivity, or causal interaction.
- Selected-feature counts describe representation composition rather than prediction effect sizes or neurophysiological relevance. We performed permutation, ablation, fold-stability, and perturbation analyses (Table A10, Table A11 and Table A12) and montage-opportunity comparisons (Table A16). Protocol A explanations are derived from whole-dataset selection and share its data dependence. The controls do not establish frontal cortical dominance, physiological coupling, or biomarker validity.
- Naive segment Wilson intervals assume independent observations, whereas repeated segments within participants are dependent. The original-analysis beta-model intervals describe within-unit segment accuracy. Aggregated Wilson intervals do not fully capture overlapping training folds or pipeline-refitting uncertainty. TMPD aggregate intervals describe 54 identifier groups, not 55 individual decisions. Class-specific aggregated intervals are wide, particularly for the 17 TMPD high-performance identifier groups and the 10 MAT low-performance participants.
- Classifier benchmarks in Table A14 used the same outer held-out partitions and were tuned using training data only. Protocol C accuracies were 80.46%, 82.50%, and 74.65%, versus EEGNet 79.4%, 78.5%, and 71% for STEW, TMPD, and MAT. Earlier-extractor comparisons in Table A15 used Protocol A and inherit its whole-dataset selection optimism. MAT baselines were close to the 71.83% majority-class reference. The Protocol A Wilcoxon results do not test the smaller Protocol C contrasts, so no statistically established Protocol C superiority is claimed.

We characterize computational requirements using analytical construction, classifier, and storage bounds in Section 3.5. Table A17 gives the dense double-precision feature-array size for each dataset. These values quantify feature storage and exclude signal buffers, temporary arrays, and selector workspaces.

We did not benchmark wall-clock runtime or peak process memory; the computational analysis is therefore limited to analytical complexity and feature-array storage, and no efficiency advantage over deep-learning models is claimed.

#### 5.4.2. Future Work

A controlled heterogeneity analysis should document electrode coverage, artifact handling, physical segment duration, and effective band limits and evaluate a justified common-channel and sampling-rate configuration where feasible. Region and layer recurrence should be assessed against null models that respect available feature opportunities. Task-matched recordings across devices are needed to separate acquisition effects from endpoint differences. Cross-dataset transfer should use compatible endpoints and a prespecified source/target design, with the model frozen before target evaluation. Any target-domain adaptation should be reported separately. These analyses have not been performed in the present study.

Independent cohorts collected with documented devices and compatible endpoints are needed to evaluate external prediction and feature-label reproducibility. A frozen model should be evaluated separately from any procedure that refits preprocessing, feature selection, or classification on the new dataset. This distinction prevents adaptation performance from being described as unchanged cross-dataset generalization.

Future regional comparisons should report which electrodes contribute to each region and how many candidate channel pairs are available. A shared regional label cannot create missing electrode coverage or guarantee equivalent cortical sampling. Common-channel sensitivity analyses and opportunity-aware null comparisons should retain participant-separated estimation and the stated endpoint definitions.

The model-based XAI findings should be compared with expert neurological interpretation. This comparison can help evaluate whether the selected frontal, temporal, central, and rhythm-transition patterns are neurophysiologically meaningful.

Prospective studies should evaluate whether any model-derived feature associations are reproducible and have physiological or clinical relevance. Such evaluations require independent cohorts, repeated recordings, and endpoints appropriate to the proposed interpretation. Until then, the associations remain descriptive features of the model.

For further XAI validation, we recommend participant-separated folds and dependence-aware opportunity or degree-preserving null models. We interpret our fixed-selection permutation control at the classifier level. Prediction-level faithfulness and neurophysiological relevance require independent evaluation.

Future benchmarking should retain exact outer partitions, training-only tuning, stopping rules, aggregation, and participant-linked predictions. Protocol C comparisons require paired analyses distinct from the original Protocol A Wilcoxon tests. Common tuning budgets and a prespecified multiplicity procedure would strengthen confirmatory comparisons.

### 5.5. Potential Implications

BioTWavePat-XFE provides classification outputs and selected feature identities for cognitive-monitoring research. These identities identify the constructed representation and do not explain the physiological mechanisms behind a prediction.

This model may be useful for education and learning analytics. Mental performance can change during learning, testing, fatigue, or attention loss. BioTWavePat-XFE can help monitor these changes using EEG signals. In this way, personalized learning systems may be designed in future studies.

BioTWavePat-XFE may be investigated for neuroergonomics and human–computer interaction. Its explicit feature identities support inspection of the representation. Online monitoring and adaptive decision support require separate latency, memory, and prospective validation; practical deployment is not established by the present offline evaluation.

The XAI outputs may help researchers inspect selected rhythm-layer and electrode-group identities. Directed Lobish makes the computational source and target labels readable. This supports hypothesis generation while retaining the distinction between a feature association and a physiological mechanism.

Chen et al. [52] examined brain-computer interface combined with functional electrical stimulation in older stroke survivors over 4.5 years, relating EEG profiling to motor function, functional independence, and cognitive assessments. This longitudinal intervention setting illustrates how EEG observations can be assessed alongside clinically defined outcomes. It differs from the mental performance classification settings evaluated here. BioTWavePat-XFE did not evaluate rehabilitation efficacy or independently validate EEG biomarkers. Any proposed rehabilitation application therefore requires independent longitudinal assessment using relevant clinical endpoints; selected-feature associations alone cannot establish neuroplasticity or functional recovery.

Repeated frontal-label and cross-rhythm feature identities provide targets for independent reproducibility studies. Their recurrence is not sufficient to designate them as biomarkers. Physiological relevance and clinical utility require separate validation.

BioTWavePat-XFE provides a traceable EEG representation with potential research applications in cognitive assessment, educational monitoring, and neuroergonomics. Its interpretation remains at the model-derived feature-association level.

**Figure 13:**
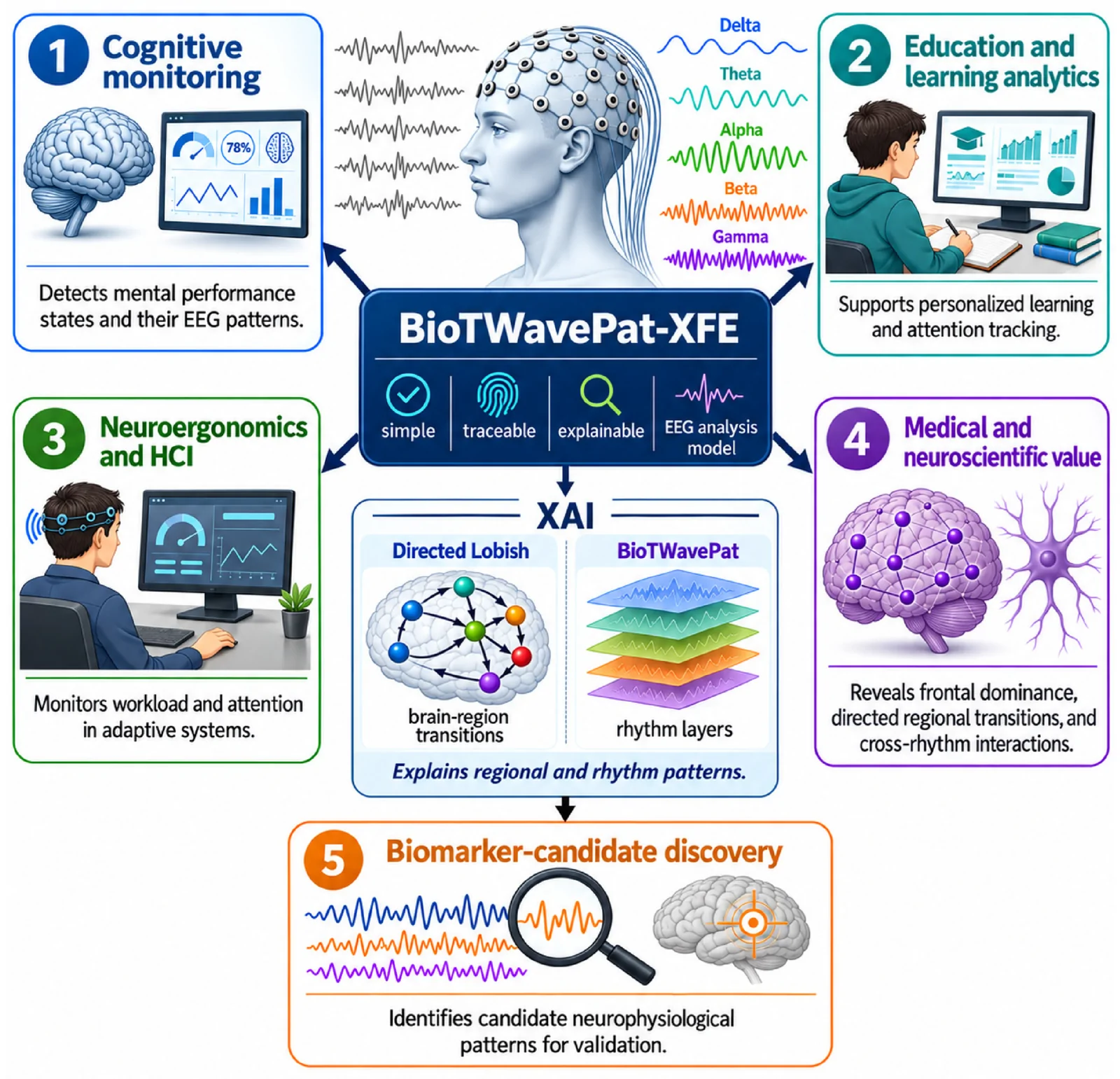
Potential implications of the proposed BioTWavePat-XFE model.

These potential implications are also summarized in Figure 13.

Figure 13 summarizes potential research applications that require further validation. Directed Lobish supports inspection of electrode-group feature identities, while BioTWavePat retains rhythm-layer identities. Physiological interpretation and clinical use are future evaluation questions; the current model-derived directed feature associations do not constitute validated biomarkers.

## 6. Conclusions

BioTWavePat-XFE combines rhythm-indexed channel-rank transition features, cumulative-weighted selection, tkNN/IMV classification, and Directed Lobish decoding. Its output includes traceable feature identities rather than validated physiological explanations.

Protocol A original segment accuracies were 83.08% (STEW), 93.14% (TMPD), and 91.15% (MAT); these estimates are subject to whole-dataset selection bias. Protocol C re-estimated all data-dependent steps using only each outer training fold and yielded accuracies of 80.46%, 82.50%, and 74.65% (balanced accuracies 80.46%, 75.17%, and 64.15%). We regard Protocol C as the primary participant-separated estimate under the stated validation design. Feature-selection leakage inflated the original results, particularly for TMPD and MAT. MAT performance was close to majority prediction and does not support meaningful discrimination. TMPD used the 54 recorded identifier groups rather than 55 separate participant folds.

Protocol C aggregated accuracies were 89.58% (43/48 STEW participants), 81.48% (44/54 TMPD identifier groups covering all 55 participants), and 75.00% (27/36 MAT participants). MAT majority prediction correctly classifies 26/36 participants. These are separate within-dataset endpoints, not frozen-model transfer across devices or tasks.

Directed Lobish summarized the identities of selected entries, including frequent FL and FR scalp labels and single-rhythm and cross-rhythm layer labels. These post hoc summaries provide traceability of the retained representation. They do not establish frontal cortical dominance, physiological rhythm interactions, connectivity, causality, or validated prediction importance. Biological and quantitative explanatory validation remain necessary.

Directed Lobish exposes selected layer and ordered electrode-label identities. Protocol A explanation sets are derived from whole-dataset selection and remain exploratory. The fixed-feature permutation control and Protocol A comparative tests do not validate the Protocol C pipeline or establish biological importance. Independent physiological and external validation are needed before clinical or biomarker use.

We report analytical computational complexity and dense feature-storage requirements for the 20C^2^ representation.

## Figures and Tables

**Table 1:** Summary of representative EEG-based mental performance, mental workload, and cognitive load detection studies.

Study	Task	Method	Classifier	Validation
Maghsoudi and Shalbaf [29]	Mental arithmetic task vs. baseline	Effective connectivity features obtained using DTF, dDTF, and GPDC with hierarchical feature selection	SVM	The best accuracy was 89.00% using GPDC and concave minimization in the beta2 band
Zhou et al. [30]	Cross-task cognitive workload recognition using working memory and mathematical addition tasks	EEG-based domain adaptation features	SVM with domain adaptation	The proposed task transfer framework improved mean accuracy by 3–8% over the non-transfer classifier
Siddhad et al. [31]	STEW and a local raw EEG dataset	Raw EEG representation	Transformer network	95.28% accuracy for two workload levels and 88.72% accuracy for three workload levels on STEW
Ajra et al. [32]	Mental arithmetic task	Spectral-temporal EEG features	Shallow CNN	The highest classification accuracy was 90.68%
Nguyen et al. [33]	Three-level mental workload estimation	Time-domain TCN and frequency-domain multi-dimensional residual block representations	Multi-space deep model	74.98% accuracy for three-class classification and 0.629 CCC for continuous estimation
Aksu et al. [13]	N-back-based mental workload task	EEG and eye-tracking features with feature selection	Machine learning classifiers	76.59% accuracy for four-class mental workload classification using 34 selected features
Safari et al. [8]	STEW dataset	Effective brain connectivity features extracted using dDTF with hierarchical feature selection	SVM, LDA, random forest, and decision tree	The best result was 89.53% ± 1.36 accuracy using SVM and forward feature selection
Gangapuram and Manian [34]	Rest vs. mental arithmetic working memory task	Bayesian functional connectivity features	Graph convolutional network	The highest subject-specific accuracy was 98%, and the average accuracy was 91% in the Beta band
Afzal et al. [35]	STEW dataset	STFT-based frequency-domain EEG representation	Deep gated neural network based on Bi-LSTM and GRU	99.45% accuracy for cognitive workload monitoring
Yedukondalu et al. [11]	MAT and STEW datasets	R-LMD-based multi-domain lead-wise EEG features with BAO-based feature optimization	Optimizable ensemble learning	97.40% accuracy on MAT and 96.10% accuracy on STEW
Siddhad et al. [36]	STEW dataset	Deep EEG representation	ConvNeXt-based neural network	95.76% accuracy for binary classification and 95.11% accuracy for multi-class classification

**Table 2:** Dataset characteristics. TMPD includes 55 participants under 54 recorded identifiers; the shared same-class identifier was held out as one group.

Dataset	Task/Condition	Participants/Held-Out Units	EEG Acquisition	Class Definition	Reference
STEW	SIMKAP multitasking workload	48 participants; 48 held-out units	Emotiv EPOC, 14 channels, 128 Hz	Participant self-report split: 24 low, 24 high	[20]
MAT	Serial subtraction arithmetic	36 participants; 36 held-out units	Neurocom, 19 EEG channels (10–20), 500 Hz	Bad/good arithmetic performance	[21]
TMPD	Mental capacity test, 60 questions	55 included participants; 54 recorded identifier groups	Emotiv Flex 2 Saline, 32 channels, 256 Hz	Low <90; high ≥90; 37 low/17 high identifier groups	[22]

**Table 3:** Segment-level EEG sample distribution used in the experiments.

Dataset	Total EEG Segments	Class 0 (Low) Segments	Class 1 (High) Segments	Distribution Property
STEW	3552	1776	1776	Balanced
TMPD	3949	2748	1201	Imbalanced
MAT	1594	449	1145	Imbalanced

**Table 4:** Segment-level outer-held-out metrics for three selection protocols. STEW/MAT use participant LOSO; TMPD uses 54 identifier-group folds covering all 55 participants. Protocol B retains whole-dataset feature selection.

Dataset/Protocol	Accuracy (%)	BA (%)	Macro-F1 (%)	Sens. C1 (%)	Spec. C0 (%)	MCC
STEW/A	83.08	83.08	83.06	86.54	79.62	0.663
STEW/B	82.35	82.35	82.32	85.98	78.72	0.649
STEW/C	80.46	80.46	80.45	83.22	77.70	0.610
TMPD/A	93.14	89.47	91.45	80.10	98.84	0.837
TMPD/B	92.53	88.56	90.65	78.43	98.69	0.822
TMPD/C	82.50	75.17	77.22	56.45	93.89	0.564
MAT/A	91.15	87.28	88.65	96.16	78.40	0.776
MAT/B	90.78	86.54	88.09	96.24	76.84	0.766
MAT/C	74.65	64.15	65.23	88.21	40.09	0.320

**Table 5:** Aggregated decision accuracy, class-aware metrics and descriptive 95% Wilson intervals. STEW/MAT: participants; TMPD: 54 identifier groups covering 55 participants.

Dataset	Protocol	Correct/Total	Accuracy (%)	95% Wilson CI (%)	BA (%)	Macro-F1 (%)	Sens. C1 (%)	Spec. C0 (%)	Decision Unit
STEW	A	45/48	93.75	83.16–97.85	93.75	93.75	95.83	91.67	Participant
STEW	B	44/48	91.67	80.45–96.71	–	–	–	–	Participant
STEW	C	43/48	89.58	77.83–95.47	89.58	89.58	87.50	91.67	Participant
TMPD	A	51/54	94.44	84.89–98.09	91.18	93.21	82.35	100.00	Identifier group
TMPD	B	51/54	94.44	84.89–98.09	–	–	–	–	Identifier group
TMPD	C	44/54	81.48	69.16–89.62	73.77	75.89	52.94	94.59	Identifier group
MAT	A	34/36	94.44	81.86–98.46	90.00	92.59	100.00	80.00	Participant
MAT	B	34/36	94.44	81.86–98.46	–	–	–	–	Participant
MAT	C	27/36	75.00	58.93–86.25	70.38	69.75	80.77	60.00	Participant

Table 5 note: Class-specific aggregated metrics are reported for Protocols A and C. BA denotes balanced accuracy; Sens. C1 is sensitivity, and Spec. C0 is specificity. Accuracy intervals are descriptive 95% Wilson intervals.

**Table 6:** Protocol A component performance.

Dataset	Best Single Selector	Single-Selector Accuracy Range (%)	Selected Feature Range	Best Binary Intersection	Intersection Accuracy (%)	Best IMV Accuracy (%)	Reported Final Outcome
STEW	CWChi2	75.42–80.94	75–330	CWChi2 ∩ CWmRMR	78.91	83.08	IMV-based voted outcome, 83.08%
TMPD	CWmRMR	83.44–91.24	10–224	CWChi2 ∩ CWmRMR	88.35	93.14	IMV-based voted outcome, 93.14%
MAT	CWmRMR	69.70–91.15	50–280	CWChi2 ∩ CWmRMR	82.56	90.65	tkNN-based individual outcome, 91.15%

**Table 7:** Protocol A selected feature identities derived from whole-dataset selection.

Dataset	Weighted Feature Count	Unique Feature Count	Frequent Rhythm Labels	Frequent DLob Labels	Frequent Label Pairs
STEW	1174	731	delta (20.19%), gamma-delta (16.10%), gamma (10.73%)	FR (33.09%), FL (21.89%), TR (9.03%)	FR → FR (14.91%), FR → FL (8.01%), FL → FR (7.84%)
TMPD	126	110	beta (9.52%), gamma (9.52%), thetatheta (9.52%), gamma-delta (7.94%)	FL (23.41%), FR (12.70%), CR (11.11%)	FL → FL (5.56%), FL → CR (4.76%), FR → FL (4.76%)
MAT	280	280	gamma-delta (10.36%), gamma-thetatheta (10.00%), alpha (8.57%)	FL (14.29%), TL (14.29%), FR (13.93%)	FL → FR (3.57%), FL → TR (3.21%), FR → TR (2.86%)

**Table 8:** Cross-dataset recurrence of selected feature-label associations.

DLob Transition	Shared Datasets	Summed Normalized Weight	Feature-Label Association
FR → FL	3	1.894	Frontal cross-lateral label association
FL → TR	2	1.546	Fronto-temporal label association
FL → FR	2	1.526	Frontal cross-lateral label association
FL → CR	2	1.357	Fronto-central label association
PR → FR	2	0.820	Parieto-frontal label association
TL → FL	2	0.814	Temporo-frontal label association

**Table 9:** Published EEG classification results stratified by validation protocol and prediction level.

Dataset	Study	Method/Classifier	Validation Protocol	Reported Accuracy (%) and Prediction Level	Other Reported Metrics/Qualification
A. Participant-separated evaluation: reported LOSO and this study’s TMPD identifier-group validation					
STEW	This study/A	BioTWavePat rhythm-transition features + CWNCA/CWChi2/CWmRMR/CWReliefF + tkNN + IMV + Directed Lobish Classifier: tkNN/IMV	Protocol A: whole-dataset feature selection and pooled LOSO outcome choice	83.08 segment/93.75 aggregated participant	Binary F1: 83.65 segment/93.88 participant
STEW	This study/C	BioTWavePat + training-fold CW selectors + tkNN/IMV; training-fold inner outcome validation	Protocol C: all data-dependent steps fitted only on outer-training participants	80.46 segment/89.58 aggregated participant	Segment BA 80.46; macro-F1 80.45; participant-separated outer-test estimate; participant-level self-report labels
MAT	This study/A	BioTWavePat rhythm-transition features + CWNCA/CWChi2/CWmRMR/CWReliefF + tkNN + Directed Lobish Classifier: tkNN	Protocol A: whole-dataset feature selection and pooled LOSO outcome choice	91.15 segment/94.44 aggregated participant	Binary F1: 93.98 segment/96.30 participant
MAT	This study/C	BioTWavePat + training-fold CW selectors + tkNN/IMV; training-fold inner outcome validation	Protocol C: all data-dependent steps fitted only on outer-training participants	74.65 segment/75.00 aggregated participant	Segment BA 64.15; macro-F1 65.23; participant-separated outer-test estimate;
TMPD	This study/A	BioTWavePat rhythm-transition features + CWNCA/CWChi2/CWmRMR/CWReliefF + tkNN + IMV + Directed Lobish Classifier: tkNN/IMV	Protocol A: whole-dataset feature selection; pooled 54-group outcome choice	93.14 segment/94.44 aggregated identifier group	Binary F1: 87.65 segment/90.32 participant
TMPD	This study/C	BioTWavePat + training-fold CW selectors + tkNN/IMV; training-fold inner outcome validation	Protocol C: all data-dependent steps fitted only on outer-training identifier groups	82.50 segment/81.48 aggregated identifier group	Segment BA 75.17; macro-F1 77.22; participant-separated outer-test estimate;
MAT	Baygin et al., 2023 [49]	Quantum rhombus lattice pattern + triangle pooling Classifier: SVM	Reported LOSO CV	93.40	G-mean: 88.44
TMPD	Ince et al., 2025 [22]	Cubic Pattern 3D feature extraction Classifier: tkNN	Reported LOSO CV	87.79	F1-score: 91.58
TMPD	Ince et al., 2026 [47]	Multi-strided deterministic MountPat transformation Classifier: tkNN	Reported LOSO CV	86.62	Not reported
TMPD	Ince et al., 2026 [48]	Difference-based feature extraction, DiffPat Classifier: kNN	Reported LOSO CV	84.48	Not reported
B. Participant-grouped 10-fold evaluation					
TMPD	Ince et al., 2026 [48]	Difference-based feature extraction, DiffPat Classifier: kNN	Subject-aware 10-fold; all segments of each participant in one fold	99.87	Not reported; participant grouping differs from LOSO
C. Explicitly sample-wise 10-fold evaluation					
STEW	Ince et al., 2026 [47]	Graph-based deterministic MountPat feature extraction Classifier: tkNN	Sample-wise 10-fold CV	98.20	Not reported
MAT	Ince et al., 2026 [47]	Graph-based deterministic MountPat feature extraction Classifier: tkNN	Sample-wise 10-fold CV	99.94	Not reported for this protocol
TMPD	Ince et al., 2026 [47]	Multi-strided deterministic MountPat transformation Classifier: tkNN	Sample-wise 10-fold CV	100.00	Not reported
D. Other reported k-fold evaluation					
STEW	Siddhad et al., 2024 [36]	Deep CNN specifically adapted for EEG signals Classifier: ConvNeXt	Reported 5-fold CV	95.76 binary	95.11 multi-class accuracy
STEW	Afzal et al., 2024 [35]	STFT-based frequency representation Classifier: DGNN, Bi-LSTM + GRU	Reported 5-fold CV	99.45	F1-score: 0.98–1.00 (reported scale)
STEW	Safari et al., 2024 [8]	dDTF effective connectivity + hierarchical feature selection Classifier: SVM	Reported 7-fold CV	89.53 ± 1.36	Not reported
STEW	Yedukondalu et al., 2025 [11]	R-LMD decomposition + BAO feature optimization Classifier: Optimized Ensemble Learning	Reported 10-fold CV	96.10	Not reported
STEW	Jain et al., 2024 [50]	VMD-decomposed entropy features Classifier: LightGBM	Reported 10-fold CV	95.51	F1-score: 95.71
MAT	Baygin et al., 2023 [49]	Quantum rhombus lattice pattern + triangle pooling Classifier: SVM	Reported 10-fold CV	97.88	G-mean: 96.42
MAT	Jain et al., 2024 [50]	VMD-decomposed entropy features Classifier: LightGBM	Reported 10-fold CV	97.22	F1-score: 97.43
MAT	Yedukondalu et al., 2025 [11]	R-LMD decomposition + BAO feature optimization Classifier: Optimized Ensemble Learning	Reported 10-fold CV	97.40	Not reported
TMPD	Ince et al., 2025 [22]	Cubic Pattern 3D feature extraction Classifier: tkNN	Reported 10-fold CV	99.70	Not reported for this protocol
E. Hold-out or split-based evaluation					
STEW	Siddhad et al., 2022/2024 [31]	Raw EEG classification without handcrafted feature extraction Classifier: Transformer	Reported split-based evaluation	95.28 binary/88.72 three-class	Not reported
MAT	Varshney et al., 2021 [51]	Slope, sample, approximation, dispersion, and permutation entropy features Classifier: RNN, LSTM, BLSTM, GRU	Reported hold-out CV	99.81	F1-score: 99.61
F. Unresolved validation-protocol reporting					
MAT	Ince et al., 2026 [47]	Graph-based deterministic MountPat feature extraction Classifier: tkNN	Reported subject-independent/LOSO comparison; conflicting LOSO availability statements	76.77	Macro-F1: 75.23; protocol consistency unresolved

Table 9 notes: Protocol groups describe the reported validation design, not a shared experimental benchmark. Group D does not establish participant isolation from the fold count; Group E does not establish it from a hold-out or split label. For third-party results, the prediction unit and averaging rule are those of the cited study and are not equated with the aggregated participant decisions in this work. F1 values retain their reported scale and averaging convention; binary F1 is not interchangeable with macro-F1. No between-study significance test or direct superiority claim is made. Abbreviations: EEG, electroencephalography; STEW, Simultaneous Task EEG Workload; MAT, mental arithmetic task; TMPD, Turkish Mental Performance Detection; BioTWavePat, Biological Turker Wave Pattern; XFE, explainable feature engineering; CWNCA, cumulative weighted neighborhood component analysis; CWChi2, cumulative weighted chi-square; CWmRMR, cumulative weighted minimum redundancy maximum relevance; CWReliefF, cumulative weighted ReliefF; NCA, neighborhood component analysis; Chi2, chi-square; mRMR, minimum redundancy maximum relevance; tkNN, t-algorithm-based k-nearest neighbor; kNN, k-nearest neighbor; IMV, iterative majority voting; LOSO CV, leave-one-subject-out cross-validation; CV, cross-validation; CNN, convolutional neural network; ConvNeXt, convolutional network next; STFT, short-time Fourier transform; DGNN, deep gated neural network; Bi-LSTM, bidirectional long short-term memory; LSTM, long short-term memory; BLSTM, bidirectional long short-term memory; GRU, gated recurrent unit; dDTF, direct directed transfer function; SVM, support vector machine; R-LMD, robust local mean decomposition; BAO, binary arithmetic optimization; VMD, variational mode decomposition; LightGBM, light gradient boosting machine; RNN, recurrent neural network; DLob, Directed Lobish; INCA, iterative neighborhood component analysis; DiffPat, Different Pattern; MountPat, Mountain Pattern; G-mean, geometric mean; F1-score, harmonic mean of precision and recall; F1-macro, macro-averaged F1-score; 3D, three-dimensional.

## Data Availability

The datasets used and/or analyzed during the current study are available from the corresponding author on reasonable request.

## Abbreviations

The following abbreviations are used in this manuscript:

EEG	electroencephalography
STEW	Simultaneous Task EEG Workload
MAT	mental arithmetic task
TMPD	Turkish Mental Performance Detection
BioTWavePat	Biological Turker Wave Pattern
XFE	explainable feature engineering
XAI	Explainable Artificial Intelligence
AI	Artificial Intelligence
CWNCA	cumulative weighted neighborhood component analysis
CWChi2	cumulative weighted chi-square
CWmRMR	cumulative weighted minimum redundancy maximum relevance
CWReliefF	cumulative weighted reliefF
NCA	neighborhood component analysis
Chi2	chi-square
mRMR	minimum redundancy maximum relevance
tkNN	t-algorithm-based k-nearest neighbor
kNN	k-nearest neighbor
IMV	iterative majority voting
LOSO CV	leave-one-subject-out cross-validation
CV	cross-validation
CNN	convolutional neural network
ConvNeXt	convolutional network next
STFT	short-time Fourier transform
DGNN	deep gated neural network
Bi-LSTM	bidirectional long short-term memory
LSTM	long short-term memory
BLSTM	bidirectional long short-term memory
GRU	gated recurrent unit
dDTF	direct directed transfer function
SVM	support vector machine
R-LMD	robust local mean decomposition
BAO	binary arithmetic optimization
VMD	variational mode decomposition
LightGBM	light gradient boosting machine
RNN	recurrent neural network
DLob	Directed Lobish
INCA	iterative neighborhood component analysis
DiffPat	Different Pattern
MountPat	Mountain Pattern
G-mean	geometric mean
F1-score	harmonic mean of precision and recall
F1-macro	macro-averaged F1-score
3D	three-dimensional

## Appendix A

### Appendix A.1. Additional Experimental Results

Table A1, Table A2, Table A3 and Table A4 retain Protocol A selector, intersection, and voting results. The original final outcome was the highest pooled LOSO accuracy, not the result of an inner LOSO search.

**Table A1:** Protocol A selector-wise segment outer-held-out accuracies. STEW/MAT: participant LOSO; TMPD: 54 identifier-group folds.

Dataset	CWNCA (%)	CWChi2 (%)	CWmRMR (%)	CWReliefF (%)	Best Single Selector
STEW	75.73	80.94	80.38	75.42	CWChi2
TMPD	86.73	85.57	91.24	83.44	CWmRMR
MAT	69.70	81.05	91.15	74.65	CWmRMR

**Table A2:** Number of selected features obtained by each cumulative weighted selector. Protocol A; estimates are subject to whole-dataset selection bias.

Dataset	CWNCA	CWChi2	CWmRMR	CWReliefF
STEW	75	216	329	330
TMPD	10	224	100	37
MAT	65	50	280	160

**Table A3:** Best binary-intersection results. Protocol A; estimates are subject to whole-dataset selection bias.

Dataset	Best Binary Intersection	Accuracy (%)
STEW	CWChi2-CWmRMR	78.91
TMPD	CWChi2-CWmRMR	88.35
MAT	CWChi2-CWmRMR	82.56

**Table A4:** Effect of IMV decision fusion. Protocol A; estimates are subject to whole-dataset selection bias.

Dataset	Best Individual tkNN (%)	Best IMV (%)	Reported Final Accuracy (%)	Final Outcome Type
STEW	80.94	83.08	83.08	IMV-based voted outcome
TMPD	91.24	93.14	93.14	IMV-based voted outcome
MAT	91.15	90.65	91.15	tkNN-based individual outcome

### Appendix A.2. Additional Classification Metrics

Table A5 and Table A6 retain Protocol A outcome types and positive-class metrics. Table 4 compares three protocols, Table 5 compares aggregated accuracy, and Table A18 retains the original class-aware and supplementary metrics.

**Table A5:** Additional Protocol A segment-level outer-held-out metrics.

Dataset	Reported Outcome Type	Accuracy (%)	Precision (%)	Recall (%)	G-Mean (%)	F1-Score (%)
STEW	IMV-based voted outcome	83.08	80.94	86.54	83.01	83.65
TMPD	IMV-based voted outcome	93.14	96.78	80.10	88.98	87.65
MAT	tkNN-based individual outcome	91.15	91.90	96.16	86.82	93.98

Table A5 reports Class 1 precision, recall, and F1-score; the geometric mean combines Class 1 sensitivity and Class 0 specificity. The outcome type identifies the prediction rule used in the reported evaluation. These estimates are subject to whole-dataset selection bias under Protocol A.

**Table A6:** Additional Protocol A aggregated-decision metrics. STEW/MAT: participants; TMPD: identifier groups.

Dataset	Reported Outcome Type	Aggregated Accuracy (%)	Precision (%)	Recall (%)	G-Mean (%)	F1-Score (%)
STEW	IMV-based voted outcome	93.75	92.00	95.83	93.73	93.88
TMPD (identifier groups)	IMV-based voted outcome	94.44	100.00	82.35	90.75	90.32
MAT	tkNN-based individual outcome	94.44	92.86	100.00	89.44	96.30

Table A6 reports Class 1 precision, recall, and F1-score; the geometric mean combines Class 1 sensitivity and Class 0 specificity. The outcome type identifies the prediction rule used in the reported evaluation. These estimates are subject to whole-dataset selection bias under Protocol A.

### Appendix A.3. Novelty Comparison

Table A7 compares the mathematical feature representations and published application settings. All dimensions refer to stored features before selection; C is the number of EEG channels. Earlier-study application settings are contextual descriptions, not experimental controls for the present study.

**Table A7:** Dedicated novelty comparison with earlier pattern-based EEG frameworks.

Method	Mathematical Construction	Stored Dimension	Experimental Context/Novelty Boundary
ChannelPat/Lobish [18]	Pair coding of amplitude-ranked channel identities, followed by histogram extraction. Lobish decodes selected identities into lobe symbols.	C^2^ 14: 196 32: 1024	EEG artifact classification. Channel ranking, pair encoding, and symbolic decoding are prior components; Lobish itself is a decoder.
TTPat/Directed Lobish [19]	Channel-based transformation and transition-table features; Directed Lobish adds lobe and hemisphere information to feature decoding.	392 reported for 14 channels; general formula not asserted here.	EEG artifact classification across 30 cases. Transition tables and Directed Lobish are prior components.
MountPat [47]	Five sample-vector maps and 10 pair-difference maps; ranked channels produce 15 transition tables.	15C^2^ 14: 2940 32: 15,360	EEG mental performance/arithmetic tasks. Pair-difference maps and layered transition tables are prior components; BioTWavePat uses rhythm-filtered maps.
CubicPat [22]	Ranked channel triplets (a, b, c) are encoded as (a − 1)C^2^ + (b − 1)C + (c − 1); a histogram stores triplet frequencies.	C^3^ 14: 2744 32: 32,768	Mental performance and stress classification. Three-channel identities differ from BioTWavePat’s layer-indexed channel pairs.
DiffPat [48]	Consecutive sample vectors are ranked; changed channel identities at corresponding rank positions increment a channel-pair table.	C^2^ 14: 196 32: 1024	Mental performance classification. Consecutive-sample rank changes differ from BioTWavePat’s filtered rhythm-layer bank.
BioTWavePat (this study)	Five filtered rhythm and 15 signed difference maps; normalized C × C rank-transition tables; each feature retains (layer, source, target).	20C^2^ 14: 3920 32: 20,480	STEW/MAT participant LOSO; TMPD 54-group validation including all 55 participants. Protocol A ablations and extractor benchmarks are reported. The novelty is the rhythm-indexed representation.

Table A7 compares stored representations. The rhythm/channel ablations in Table A11 and earlier-extractor experiments in Table A15 were conducted under Protocol A. Representation length is not a measure of feature independence or evidence of superiority under Protocol C.

### Appendix A.4. Class-Specific Aggregated Uncertainty

Table A8 reports class-recall intervals under Protocols A and C based on Figure 7. STEW and MAT denominators are participants; TMPD denominators are recorded identifier groups. Class 0 recall is specificity, and Class 1 recall is sensitivity. Each denominator includes only units from that class.

**Table A8:** Protocol A and Protocol C class recalls with descriptive 95% Wilson intervals. STEW/MAT: participants; TMPD: identifier groups.

Dataset	Protocol	Class 0 Correct/Total	Class 0 Recall (%) (95% CI)	Class 1 Correct/Total	Class 1 Recall (%) (95% CI)
STEW	A	22/24	91.67 (74.15–97.68)	23/24	95.83 (79.76–99.26)
STEW	C	22/24	91.67 (74.15–97.68)	21/24	87.50 (69.00–95.66)
TMPD (identifier groups)	A	37/37	100.00 (90.59–100.00)	14/17	82.35 (58.97–93.81)
TMPD (identifier groups)	C	35/37	94.59 (82.30–98.50)	9/17	52.94 (30.96–73.83)
MAT	A	8/10	80.00 (49.02–94.33)	26/26	100.00 (87.13–100.00)
MAT	C	6/10	60.00 (31.27–83.18)	21/26	80.77 (62.12–91.49)

Table A8 shows broad class-specific uncertainty, particularly for the 10 MAT Class 0 participants and 17 TMPD Class 1 identifier groups. The intervals do not account for overlapping training folds, pipeline refitting, or multiplicity. They do not measure variation in segment accuracy within a participant or group.

### Appendix A.5. Theoretical Feature Storage

Table A9 illustrates the dense payload implied by d = 20C^2^, using double precision (8 bytes per entry). The 14- and 32-channel rows correspond to the channel counts of STEW and TMPD; 64 and 128 channels are hypothetical scaling examples. *N* = 1000 is an illustrative common segment count, not a reported dataset size. KiB and MiB use 1024 and 1,048,576 bytes, respectively.

**Table A9:** Theoretical dense feature payload before selection.

Channels C	Features d = 20C^2^	One Segment (KiB)	1000 Segments (MiB)
14	3920	30.625	29.907
32	20,480	160.000	156.250
64	81,920	640.000	625.000
128	327,680	2560.000	2500.000

Table A9 describes feature-array payload only; it excludes EEG buffers, ranked sequences, selectors, training records, model objects, and allocator overhead. Doubling C quadruples both d and the dense payload. Layer-at-a-time processing can reduce simultaneous signal buffers, while retaining all feature entries still requires 20C^2^ values per segment. Total process memory also depends on the implementation and its working arrays.

### Appendix A.6. Supplementary Reviewer-Requested Empirical Analyses

We performed the supplementary experiments in Table A10, Table A11, Table A12, Table A13, Table A14, Table A15, Table A16, Table A17, Table A18, Table A19 and Table A20. Protocols B and C are reported alongside Protocol A in Table 4 and Table 5. Ablations, perturbation tests, earlier-extractor comparisons, and Wilcoxon comparisons used Protocol A; fold-wise feature-set stability used Protocol C. Classifier baselines used the same outer partitions with training-only tuning.

**Table A10:** Conditional classifier-level participant-label permutation control with fixed feature selection (T05). This is not a full-pipeline significance test.

Statistic/Design	STEW	TMPD	MAT
Participant-label permutations	100	100	100
Feature selection	Fixed	Fixed	Fixed
Corrected Monte Carlo p	0.0099	0.0099	0.0099
Inferential scope	Conditional classifier control	Conditional classifier control	Conditional classifier control

We held feature selection fixed during the participant-label permutation control and obtained a corrected Monte Carlo *p*-value of 0.0099 for each dataset.

**Table A11:** Protocol A rhythm/channel and selection ablations (T06), segment accuracy (%).

Ablation Configuration	STEW	TMPD	MAT
Protocol A complete model (biased reference)	83.08	93.14	91.15
Single-rhythm layers only (5 layers)	79.1	90.2	86.45
Cross-rhythm layers only (15 layers)	81.95	92.3	90.4
Remove five delta-associated layers	79.6	92.4	89.85
Remove FL/FR channels	76.8	91.3	89.1
No band/rhythm decomposition (one raw layer)	72.8	87.4	80.5
No feature selection (all 20C^2^ entries)	71.4	85.6	72.1

Table A11 compares single-rhythm, cross-rhythm, delta-removal, frontal-removal, no-decomposition, and no-selection configurations under Protocol A. These contrasts describe the original selection setting and cannot establish a fully fold-internal component benefit.

**Table A12:** Protocol C fold-wise selection stability (T07.01–12), Protocol A cross-dataset profile correlations (T07.13–18), and Protocol A perturbation controls (T08).

Measure	STEW	TMPD	MAT
Mean fold-wise selected-feature Jaccard	0.71	0.63	0.58
Fold-versus-pooled rhythm Spearman ρ	0.94	0.82	0.85
Fold-versus-pooled region Spearman ρ	0.97	0.86	0.88
Dominant-layer fold agreement (%)	95.83	59.26	66.67
Global channel relabeling: Δ accuracy (pp)	0	0	0
Participant-wise randomized channel order: accuracy (%)	57.5	66	64.5
Phase-randomized surrogate EEG: accuracy (%)	74.5	87	82

We computed fold-wise Jaccard, profile correlation, and dominant-layer agreement from Protocol C selected feature sets. Cross-dataset profile correlations were calculated from Protocol A selected-feature counts in Figure 8, Figure 9 and Figure 10: rhythm Spearman ρ = 0.36 (STEW–MAT), 0.699 (STEW–TMPD), 0.214 (MAT–TMPD); eight-region values were 0.287, 0.714, and 0.108. Protocol A global relabeling changed accuracy by zero, as expected because a consistent column permutation preserves kNN distances and selector identities up to relabeling. Participant-wise channel randomization and phase-randomized surrogates also used Protocol A. Surrogate accuracies remained 74.5%, 87%, and 82%, suggesting a substantial spectral-power contribution rather than a phase-specific association.

**Table A13:** Protocol A uncertainty, held-out-unit segment dispersion and Wilcoxon-versus-EEGNet comparisons (T09–T11). STEW/MAT units are participants; TMPD units are identifier groups.

Metric	STEW	TMPD	MAT
Protocol A segment accuracy (%)	83.08	93.14	91.15
Protocol A naive segment Wilson CI (%)	81.81–84.28	92.31–93.88	89.66–92.45
Protocol B naive segment Wilson CI (%)	81.06–83.57	91.67–93.31	89.26–92.10
Protocol C naive segment Wilson CI (%)	79.13–81.73	81.29–83.66	72.46–76.73
Protocol A aggregated Clopper–Pearson CI (%)	82.8–98.69	84.61–98.84	81.34–99.32
Protocol A beta-model cluster CI (%)	78.2–87.96	88.12–98.16	85.27–97.03
Protocol A mean held-out-unit segment accuracy (%)	83.08	93	91.2
Protocol A held-out-unit segment accuracy SD (points)	17.2	18.5	18
Protocol A median held-out-unit segment accuracy (%)	Not reported	100	97.7
Protocol A Wilcoxon versus EEGNet p	0.0021	0.0009	0.031

Table A13 reports naive segment Wilson, aggregated Clopper–Pearson, and beta-model within-unit accuracy intervals for Protocol A. STEW contributed 74 segments per participant; TMPD and MAT segment counts varied. Mean and standard deviation are reported for all three datasets; median accuracy is reported for TMPD and MAT. Wilcoxon comparisons against EEGNet used paired Protocol A held-out-unit accuracies; their *p*-values do not test Protocol C performance. McNemar counts and continuity-corrected statistics are reported in Table A19.

**Table A14:** Classifier benchmark segment accuracies (T12, %) alongside Protocol A and Protocol C.

Classifier/Protocol	STEW	TMPD	MAT
BioTWavePat/A (biased)	83.08	93.14	91.15
BioTWavePat/C	80.46	82.50	74.65
PSD band power + SVM	74.6	76.2	69.3
EEGNet	79.4	78.5	71
ShallowConvNet	77.8	77.1	70.2
DeepConvNet	76.1	75.3	71.8
EEG-Conformer	78.6	77.9	70.6
Majority-class reference	50	69.59	71.83

Classifier baselines were run on the same outer held-out partitions and tuned using training data only, including the same 54 identifier groups for TMPD. EEGNet balanced accuracies were 79.4%, 70.5%, and 55.8% for STEW, TMPD, and MAT. Protocol C accuracies of 80.46%, 82.50%, and 74.65% are compared with EEGNet 79.4%, 78.5%, and 71%. MAT baseline values were close to the 71.83% majority-class reference. These point estimates are not accompanied by a paired Protocol C significance test.

**Table A15:** Protocol A earlier pattern-extractor benchmarks (T13), segment accuracy (%).

Extractor	STEW	TMPD	MAT
Protocol A complete model (biased reference)	83.08	93.14	91.15
TTPat	79.2	88.4	86.2
LobishPat	77.9	87	84.9
CubicPat	80.1	89.3	88
MountPat	80.6	88.6	79.5
DiffPat	78.7	86.9	85.4

The TTPat, LobishPat, CubicPat, MountPat, and DiffPat experiments used the CW-selector/tkNN/IMV framework under Protocol A. They share the original whole-dataset selection boundary and its optimism. Table A15 complements the mathematical comparison in Table A7; it does not demonstrate Protocol C superiority.

**Table A16:** (**a**). Protocol A montage-opportunity and selected-layer null analyses (T14), derived from whole-dataset selection. (**b**). Protocol A regional-pair null comparisons derived from whole-dataset selection.

(**a**)					
Measure			STEW	TMPD	MAT
Observed frontal-label fraction (%)			54.98	36.11	28.21
Montage-expected frontal fraction (%)			57.14	31.25	31.58
Frontal enrichment, one-sided p			0.9835	0.0565	0.9617
FR → FL observed/expected ratio			0.981	1.95	0.716
FR → FL enrichment p			0.593	0.089	0.829
delta-associated selected-layer share (%)			55.79	31.75	33.57
Regional goodness-of-fit χ^2^			82.54	64.02	38.32
Uniform 20-layer χ^2^ (df 19)			1189.68	40.03	77.71
Regional χ^2^ degrees of freedom			7	13	12
Approximate regional χ^2^ p			4.2 × 10^−15^	9.9 × 10^−9^	0.00014
Approximate uniform-layer χ^2^ p			1.5 × 10^−240^	0.0032	4.6 × 10^−9^
Approximate delta-share p (expected 25%)			2 × 10^−111^	0.053	0.00081
(**b**)					
Dataset/Pair	Count	Observed (%)	Expected (%)	O/E	Approx. *p*
STEW/FR → FL	94	8.0068	8.1633	0.9808	0.5929
STEW/FL → TR	13	1.1073	2.0408	0.5426	0.9948
STEW/FL → FR	92	7.8365	8.1633	0.96	0.6738
STEW/PR → FR	26	2.2147	2.0408	1.0852	0.3641
STEW/TL → FL	20	1.7036	2.0408	0.8348	0.8203
STEW/FR → FR	175	14.9063	8.1633	1.826	1.376 × 10^−14^
STEW/FL → FL	78	6.644	8.1633	0.8139	0.9773
MAT/FR → FL	5	1.7857	2.4931	0.7163	0.8287
MAT/FL → TR	9	3.2143	1.662	1.9339	0.0464
MAT/FL → FR	10	3.5714	2.4931	1.4325	0.165
MAT/FL → CR	5	1.7857	0.831	2.1488	0.0861
MAT/PR → FR	1	0.3571	0.831	0.4298	0.9033
MAT/TL → FL	7	2.5	1.662	1.5042	0.1876
MAT/FR → FR	5	1.7857	2.4931	0.7163	0.8287
MAT/FL → FL	2	0.7143	2.4931	0.2865	0.9931
TMPD/FR → FL	6	4.7619	2.4414	1.9505	0.0893
TMPD/FL → TR	4	3.1746	0.9766	3.2508	0.0357
TMPD/FL → FR	0	0	2.4414	0	1
TMPD/FL → CR	6	4.7619	1.4648	3.2508	0.011
TMPD/PR → FR	4	3.1746	0.9766	3.2508	0.0357
TMPD/TL → FL	0	0	0.9766	0	1
TMPD/FR → FR	4	3.1746	2.4414	1.3003	0.37
TMPD/FL → FL	7	5.5556	2.4414	2.2756	0.0355

We compared Protocol A selected-feature frequencies with montage opportunities. STEW frontal groups were FL = AF3, F7, F3, FC5 and FR = AF4, F8, F4, FC6, giving eight frontal electrodes among 14. MAT used the standard 19-channel 10–20 scalp grouping. TMPD had 10 frontal channels among 32. The expected frontal fractions were therefore 57.14%, 31.58%, and 31.25%, respectively. Pair-specific expected frequencies use the original DLob source/target group coding and describe that montage rather than a common anatomical sampling scheme. Selected features are dependent, so binomial and χ^2^ *p*-values are approximate and unadjusted. Table A16b lists the tested pairs without inferring physiological directionality.

Table A16b lists all additional regional pairs evaluated against the montage-opportunity null. *p*-values are approximate and unadjusted; no extra multiplicity-corrected values are computed here.

Table A16b includes all tested regional pairs. For STEW FR → FR, the observed share was 175/1174 against the montage-based expected share p_0_ = (4/14)^2^ = 0.081633, giving an observed-to-expected ratio of 1.826. The one-sided binomial upper tail P[X ≥ 175], with X ~ Binomial (1174, p_0_), was 1.376 × 10^−14^. This is an approximate, unadjusted post hoc montage comparison because selected occurrences are dependent; it is distinct from the participant-label permutation control.

**Table A17:** Analytical dense double-precision feature-array storage.

Quantity	STEW	TMPD	MAT
Segments N	3552	3949	1594
Channels C	14	32	19
Features per segment d = 20C^2^	3920	20,480	7220
Dense double feature-array storage (decimal MB)	111.39	647.00	92.07

We calculate dense feature-array storage as 8N(20C^2^) bytes, where N denotes the number of segments and C the number of EEG channels. Table A17 gives 111.39 MB for STEW, 647.00 MB for TMPD, and 92.07 MB for MAT, using decimal MB (10^6^ bytes). These sizes describe the feature arrays before selection and exclude raw EEG buffers, temporary copies, classifier objects, and selector workspaces. Doubling the number of channels quadruples the feature-array size at fixed N.

Table A18 preserves Protocol A class-aware metrics and the positive-class F1 scores of Protocols B and C.

**Table A18:** Additional class-aware performance metrics.

Protocol/Level	Metric	STEW	TMPD	MAT
A/Segment	Balanced accuracy (%)	83.08	89.47	87.28
A/Segment	Macro-F1 (%)	83.06	91.45	88.65
A/Segment	Sensitivity C1 (%)	86.54	80.1	96.16
A/Segment	Specificity C0 (%)	79.62	98.84	78.4
A/Segment	F1 C0 (%)	82.47	95.25	83.31
A/Segment	NPV (%)	85.54	91.91	88.89
A/Segment	Cohen κ	0.6616	0.8296	0.7733
A/Segment	MCC	0.6632	0.8367	0.7761
A/Aggregated participant/identifier group	Balanced accuracy (%)	93.75	91.18	90
A/Aggregated participant/identifier group	Macro-F1 (%)	93.75	93.21	92.59
A/Aggregated participant/identifier group	Sensitivity C1 (%)	95.83	82.35	100
A/Aggregated participant/identifier group	Specificity C0 (%)	91.67	100	80
A/Aggregated participant/identifier group	F1 C0 (%)	93.62	96.1	88.89
A/Aggregated participant/identifier group	NPV (%)	95.65	92.5	100
A/Aggregated participant/identifier group	Cohen κ	0.875	0.8648	0.8525
A/Aggregated participant/identifier group	MCC	0.8758	0.8728	0.8619
B/Segment	F1 C1 (%)	82.97	86.46	93.75
C/Segment	F1 C1 (%)	80.99	66.24	83.33

Table A18 separates positive-class F1 from macro-F1. The Protocol A aggregated class metrics use participants for STEW/MAT and identifier groups for TMPD.

Table A19 reports exploratory McNemar comparisons for the original Protocol A component predictions with separate discordant counts and continuity-corrected one-degree-of-freedom statistics.

**Table A19:** Protocol A McNemar comparisons using confirmed discordant counts.

Dataset/Final Versus Comparator	b	c	χ^2^ (1 df)	Nominal *p*
STEW: final IMV vs. best single selector	198	122	17.58	2.76 × 10^−5^
TMPD: final IMV vs. best single selector	137	62	27.52	1.56 × 10^−7^
MAT: final CWmRMR tkNN vs. best IMV	34	26	0.82	0.366

For Table A19, b denotes segments correctly classified by the final Protocol A outcome but incorrectly by the comparator, and c denotes the reverse. The continuity-corrected statistic is χ^2^ = (|b − c| − 1)^2^/(b + c), evaluated using one degree of freedom. TMPD yielded χ^2^ = 27.52 and nominal *p* = 1.56 × 10^−7^; STEW yielded χ^2^ = 17.58 and *p* = 2.76 × 10^−5^. For MAT, final CWmRMR tkNN versus best IMV yielded χ^2^ = 0.82 and *p* = 0.366. Dependence between segments limits these exploratory comparisons, which concern Protocol A.

Table A20 records the Protocol B segment confusion counts. Protocol B retains whole-dataset feature-selection leakage.

**Table A20:** Protocol B segment confusion counts; whole-dataset selection is retained.

Dataset	TN	FP	FN	TP
STEW	1398	378	249	1527
TMPD	2712	36	259	942
MAT	345	104	43	1102

Table A20 documents the outcome-only rerun. It is not fully leakage-free because the feature sets were selected using all participants.
